# Diphenyl diselenide protects against diabetic kidney disease through modulating gut microbiota dysbiosis in streptozotocin-induced diabetic rats

**DOI:** 10.3389/fphar.2024.1506398

**Published:** 2024-12-03

**Authors:** Xing Wang, Dongmei Long, Xingcan Peng, Jiaxuan Li, Maoting Zhou, Yu Wang, Xianghong Hu

**Affiliations:** ^1^ Department of Pharmacology, School of Pharmacy, North Sichuan Medical College, Nanchong, China; ^2^ Nanchong Key Laboratory of Disease Prevention, Control and Detection in Livestock and Poultry, Nanchong Vocational and Technical College, Nanchong, China

**Keywords:** diphenyl diselenide, diabetic nephropathy, extracellular matrix, oxidative stress, Nrf2/Keap1 signaling, gut microbiota

## Abstract

**Introduction:**

Diphenyl diselenide (DPDS) ameliorates nephropathy in streptozotocin (STZ)-induced type 1 diabetic rats by inhibiting oxidative stress and inflammatory reactions. However, it has not been clarified whether DPDS alleviates type 1 diabetic kidney disease (DKD) is related to the inhibition of extracellular matrix (ECM) production and the regulation of intestinal flora disorder.

**Methods:**

The present study investigated the effects of DPDS on ECM generation in the kidney and intestinal microflora composition in feces. The rats were orally administered DPDS or metformin for eight weeks. Various indices were measured to assess the severity of renal injury. After euthanizing the rats, oxidative stress markers in serum and kidney were assessed using biochemical methods, and the expressions of ECM-related proteins in kidney were analyzed using Western blot. Additionally, 16S rRNA high-throughput sequencing was used to evaluate the diversity and composition of the intestinal flora in feces.

**Results:**

The results showed DPDS and metformin improved the DKD in STZ rats, as evidenced by decreased blood glucose, BUN, urine volume, urine microalbumin, urinary β2 microglobulin, and improvement of renal pathological morphology. Furthermore, DPDS intervention markedly reduced the protein expression of α-SMA, COI Ⅳ, FN, and vimentin in the kidneys. Besides, DPDS not only improved dyslipidemia in STZ diabetic rats, but also enhanced the activities of antioxidant enzymes, decreased the level of MDA in serum and kidney, and regulated the expression of proteins related to the Nrf2/Keap1 signaling pathway in the kidney. Moreover, we found that DPDS could selectively improve the relative abundance of probiotics as well as the diversity of flora, thus ameliorating the intestinal microbial composition of the STZ rats, significantly regulating the intestinal microbial homeostasis.

**Discussion:**

Overall, DPDS inhibited ECM production and improved renal pathological changes, which may be related to reducing oxidative stress damage in the kidney and improving intestinal flora imbalance, providing data support for the further development and application of DPDS in DKD.

## 1 Introduction

As one of the most serious and common microvascular complications of diabetes, diabetic kidney disease (DKD) is becoming a serious global public health problem in the 21st century and is also one of the main causes of end-stage renal disease. If diabetic patients do not control their blood glucose within a reasonable range, 30% of those with type 1 diabetes and 40% with type 2 diabetes will gradually develop DKD (Wei et al., 2023). With the improvement of living standards and lifestyle changes, the number of diabetes patients is increasing yearly, leading to a gradual increase in the prevalence of DKD. At present, the main strategies for treating DKD are to control blood glucose and blood pressure, reduce blood lipid, and inhibit the renin-angiotensin system (Kim et al., 2022). Although drugs targeting these strategies can alleviate kidney damage and delay the progression of DKD to some extent, there are still adverse reactions and a lack of specific treatment (Hu et al., 2023). Therefore, it is urgent to develop novel therapeutic medications to slow down the progress of DKD.

The pathological mechanism of DKD is complicated and oxidative stress and intestinal flora play a fundamental role in the progress of DKD. A large number of reactive oxygen species (ROS) produced by persistent hyperglycemia can activate various molecular pathways, destroy the antioxidant defense system, which ultimately promote the development of DKD (Wang and Zhang, 2024). In addition, a large number of studies showed that intestinal microflora has gradually become a key regulator to mediate the progress of DKD (Chi et al., 2021; Xu et al., 2024). Long-term hyperglycemia changes the physical and chemical properties of intestinal microenvironment, disturbs intestinal microflora and structure, reduces probiotics, increases harmful bacteria, activates renin-angiotensin system in kidney, oxidative stress and fibrosis, thus accelerating the progress of DKD (Yu et al., 2024). Furthermore, intestinal flora not only affects the metabolism of polysaccharides and short-chain fatty acids, but also the imbalance of intestinal flora will lead to the leakage of pro-inflammatory bacterial products, which leads to kidney damage (Tao et al., 2022). Moreover, kidney damage makes it difficult for a large number of metabolic wastes to be excreted from the body and enter the intestinal cavity through the intestinal wall, further aggravating the disorder of intestinal flora and forming a vicious circle (Han C et al., 2023). More importantly, the strategy of inhibiting oxidative stress and regulating intestinal flora disorder have shown promising therapeutic potential in the prevention of DKD (Hernandez et al., 2022; Pant et al., 2023; Li et al., 2018).

Diphenyl diselenide (DPDS) is one of the most representative synthetic organic selenium compounds, which has the characteristics of a simple structure, good stability, and less toxicity than inorganic selenium and most organic selenium compounds (Rosa et al., 2007). DPDS has strong glutathione peroxidase (GPX) simulation activity, participates in thioredoxin reductase catalytic reaction, and activates the nuclear factor E2 associated factor 2 (Nrf2)/Kelch-like epichlorohydrin-associated protein-1 (Keap1) signal pathway, so it has strong oxidation resistance (de Freitas and Rocha, 2011; Barbosa et al., 2017; Alhasan et al., 2023). Moreover, DPDS has been proved to have anti-inflammatory (Doleski et al., 2017), antifungal (Benelli et al., 2021), neuroprotective (Zhang et al., 2021), antitumor (Melo et al., 2013), liver protective (Fulco et al., 2020a), kidney protective (Fulco et al., 2020b), and cardiovascular protective (de Bem et al., 2008) effects in various animal models and cell models. Notably, in the rat model of type 1 diabetes induced by streptozotocin (STZ) or alloxan, and in the zebrafish model with hyperglycemia, it is found that DPDS improved the symptoms of hyperglycemia to different degrees, reduced the toxicity of STZ or alloxan, and ameliorated the oxidative damage of the liver and kidney to some extent (Barbosa et al., 2008; Dos Santos et al., 2020). In addition, we found that DPDS inhibited oxidative stress by regulating the Nrf2/Keap1 signaling pathway, thus improving diabetic peripheral neuropathy in STZ-induced diabetic rats (Wang et al., 2020). Of particular significance, we found that DPDS can obviously improve type 1 DKD by inhibiting oxidative stress and inflammatory reactions *in vivo* and *in vitro* (Wang et al., 2021; Wang et al., 2022). Nevertheless, it remains unknown whether DPDS alleviates type 1 DKD is related to the inhibition of ECM production and the regulation of intestinal flora disorder. Therefore, we investigated the effects of DPDS on ECM production and intestinal microflora composition in the STZ-induced DKD model and provided evidence for further clarifying the mechanism of DPDS in improving DKD.

## 2 Materials and methods

### 2.1 Reagents and antibodies

DPDS (C_12_H_10_Se_2_, MW: 312.13, purity: ≥98%, 180629) was purchased from Sigma (St. Louis, MO, United States). STZ (purity≥99%, S25467) and metformin (purity≥98%, B25331) were obtained from Yuanye Biotechnology Co., Ltd. Kits for determining blood urea nitrogen (BUN, C013-2-1), creatinine (C011-2-1), malondialdehyde (MDA, A003-4-1), total antioxidant capacity (T-AOC, A015-2-1), alanine aminotransferase (ALT, C009-2-1), superoxide dismutase (SOD, A001-3-2), Glutathione peroxidase (GSH-PX, A005-1-2), and aspartate aminotransferase (AST, C010-2-1) were acquired from Nanjing Jiancheng Bioengineering Institute (Nanjing, Jiangsu, China). Microalbuminuria (m-ALB, DG20949D-96T) and β2-microglobulin (β2-MG, DG20081D-96T) content detection kits were procured from Beijing Dongge Boye Biotechnology Co. Ltd (Beijing, China). High-density lipoprotein cholesterol (HDL-C, 100025738), total cholesterol (TC, 100060092), triglyceride (TG, 100060102), and low-density lipoprotein cholesterol (LDL-C, 100025748) level detection kits were purchased from Biosino Biotechnology and Science Inc. (Beijing, China). Antibodies against NADPH quinone oxidoreductase 1 (NQO1, 3187S), Keap1 (8047S), vimentin (5741S), and α-smooth muscle actin (α-SMA, 19245S) were purchased from Cell Signaling Technology (Danvers, MA, United States). Antibodies against COI Ⅳ (ab6586), Nrf2 (ab137550), heme oxygenase-1 (HO-1, ab189491), and FN (ab2413) were acquired from Abcam (Cambridge, Cambridgeshire, UK). The secondary antibody, protease and phosphatase inhibitors, bicinchoninic acid (BCA, P1511-1) protein kit, ristocetin-induced platelet aggregation (RIPA, C1055) lysis buffer, together with enhanced chemiluminescence (ECL, W0001) reagent were acquired from Applygen Technologies (Beijing, China).

### 2.2 Animals

Fifty male Sprague-Dawley (SD) rats (6–7 weeks old, 200 ± 20 g) were purchased from HFK Bio-Technology. co., LTD (Beijing, China). The company’s license number was SCXK (Jing) 2019–0008. Rats were housed on a 12-h light/dark cycle with humidity at 55% ± 10% and temperature at 23°C ± 2°C with *ad libitum* food and water. All experimental procedures were strictly conducted complying with the Guidelines for the Care and Use of Laboratory Animals (GB14925-2001 and MOST 2006a) and approved by the Experimental Animal Welfare Ethics Committee of the North Sichuan Medical College (Approval No. 2023011).

### 2.3 Experimental design

The methods of type 1 daibetic rats model formation, dissolved drug, and administration of drugs were consistent with the previously described protocol, and the dosage of STZ is 60 mg/kg (Wang et al., 2021). Four weeks after modeling, these rats were randomly divided into four groups (*n* = 10/group) and started to give drugs: (1) DKD model group (Mod); (2) positive drug metformin treatment group (Met); (3) DPDS low-dose treatment group (DPDS-L, 5 mg/kg); (4) DPDS high-dose treatment group (DPDS-H, 15 mg/kg). Rats injected with citric acid buffer served as the normal control group (Nor, *n* = 10). Subsequently, the drug was administered orally for 8 weeks. During the experiment, the weight, fasting blood glucose (FBG), and random blood glucose (RBG) of the rats were measured every week. In the final week, the urine of rats was collected using metabolic cages for 8 h. Before the formal start of the metabolic cage experiment, rats were placed in the metabolic cage and given enough feed and water to adapt for 2 days, so as to reduce the interference caused by stress reaction. Then the rats were anesthetized with 10% chloral hydrate, euthanized, and their kidney tissues were removed and weighed. One part of the tissue was fixed in 4% paraformaldehyde for hematoxylin and eosin (HE) and periodic acid-Schiff stain (PAS) staining, while the rest was frozen in the refrigerator at −80°C for biochemical indexes and protein detection. The renal index and creatinine clearance rate (Ccr) were calculated using the following formula: kidney index = kidney weight (mg)/body weight (g); Ccr = urine creatinine × urine volume (8 h)/serum creatinine ×1440/body weight.

### 2.4 Biochemical index analysis in serum

The whole blood was allowed to stand at room temperature for 2 h and then centrifuged for 10 min at 5000 r/min, 4°C. The supernatant was collected, and the contents of TG, ALT, TC, AST, LDL-C, SOD, HDL-C, GSH, MDA, T-AOC, as well as GPX were determined in accordance with the kit instructions.

### 2.5 Determination of biochemical indexes and histopathologic examination in kidney

Kidney tissue from each rat (100 mg) was homogenized with pre-cooled physiological saline (900 μL), and then centrifuged at 12000 r/min at 4°C for 10 min. The levels of TG, protein concentration, TC, SOD, T-AOC, GSH, and MDA in the supernatant were determined strictly according to the kit instructions, and the results were checked by protein concentration. Renal tissue was fixed in 4% paraformaldehyde for 24 h, embedded in paraffin, sliced with a microtome (4 μm), then stained with HE and PAS, and finally observed and photographed with a microscope (NIKON ECLIPSE E100, Nikon, Japan) to evaluate the pathological changes in the kidney. According to the swelling of renal tubular epithelial cells, the formation and shedding of necrotic tubules and casts, glomerular sclerosis and injury, the histopathological changes were evaluated according to the 0–4 grade renal tubular and glomerular injury. The criteria are as follows: 0, no injury; 1, <25%; 2, 25%–50%; 3, 50%–75%; 4, >75% (Xu et al., 2020). Selection of areas scoring were analyzed by people in a blind manner.

### 2.6 Western blot

The kidney tissue was homogenized in RIPA lysis buffer containing protease alongside phosphatase inhibitors. After 40 min of ice lysis, it was centrifuged at 12000/min, 4°C for 10 min. After the protein concentration was determined by the BCA method, the protein concentration was adjusted to the same level, and then 5× loading buffers were added and boiled for 10 min. Subsequent sodium dodecyl sulfate polyacrylamide gel electrophoresis was performed as previously described (Wang et al., 2020) and made some minor adjustments. Four rats’ kidneys were randomly selected from each group. After protein extraction, β-actin and target protein were detected. As for proteins with similar molecular weight, the target protein was directly re-electrophoresed without stripping procedure.

### 2.7 Fecal pellet collection and 16S rDNA sequencing

In the last week of administration, rats were placed in clean cages covered with sterile filter paper, with one rat per cage. After the rats excreted feces, they were quickly collected in sterile centrifuge tubes. When the weight of each sample exceeded 200 mg, the collected sterile centrifuge tube was immediately placed in liquid, then the filter paper was replaced, and the feces of the next rat were collected. Finally, all the samples were transported to the company for further testing with ice. The analysis of fecal 16S rDNA was entrusted to Shanghai Biotree Biotech Co., Ltd. (Shanghai, China) with mature technology. Briefly, after extracting the isolated DNA from each rat fecal bacterial strain with the QIAamp Fast DNA Stool Mini Kit (Qiagen, CA, United States), 16S rRNA was amplified in the hypervariable V3–V4 region, purified, and quantified. The purified amplicon was sequenced on Illumina MiSeq (PE300), and the data were analyzed accordingly. The amplified sequences were merged, and operational taxonomic units (OTUs) were categorized according to a sequence similarity of 97%. Alpha (α) diversity included chao1, Shannon, and Simpson index. Beta (β) diversity was evaluated by non-metric multidimensional scaling (NMDS) analysis. Bar plots and principal coordinate analysis (PCoA) plots were created using R software. Mann-Whitney *U* test and Kruskal–Wallis test were used for comparative analysis. The PICRUSt2 function prediction tool combined with KEGG database query was utilized to predict and analyze the function of the intestinal microflora.

### 2.8 Statistical analysis

Data were described with mean ± standard error of the mean (SEM), and analyzed using one-way analysis of variance along with Bonferroni’s correction. *p* < 0.05 were considered statistically significant.

## 3 Results

### 3.1 DPDS intervention reduced blood glucose and ameliorated renal dysfunction in DKD rats

**FIGURE 1:**
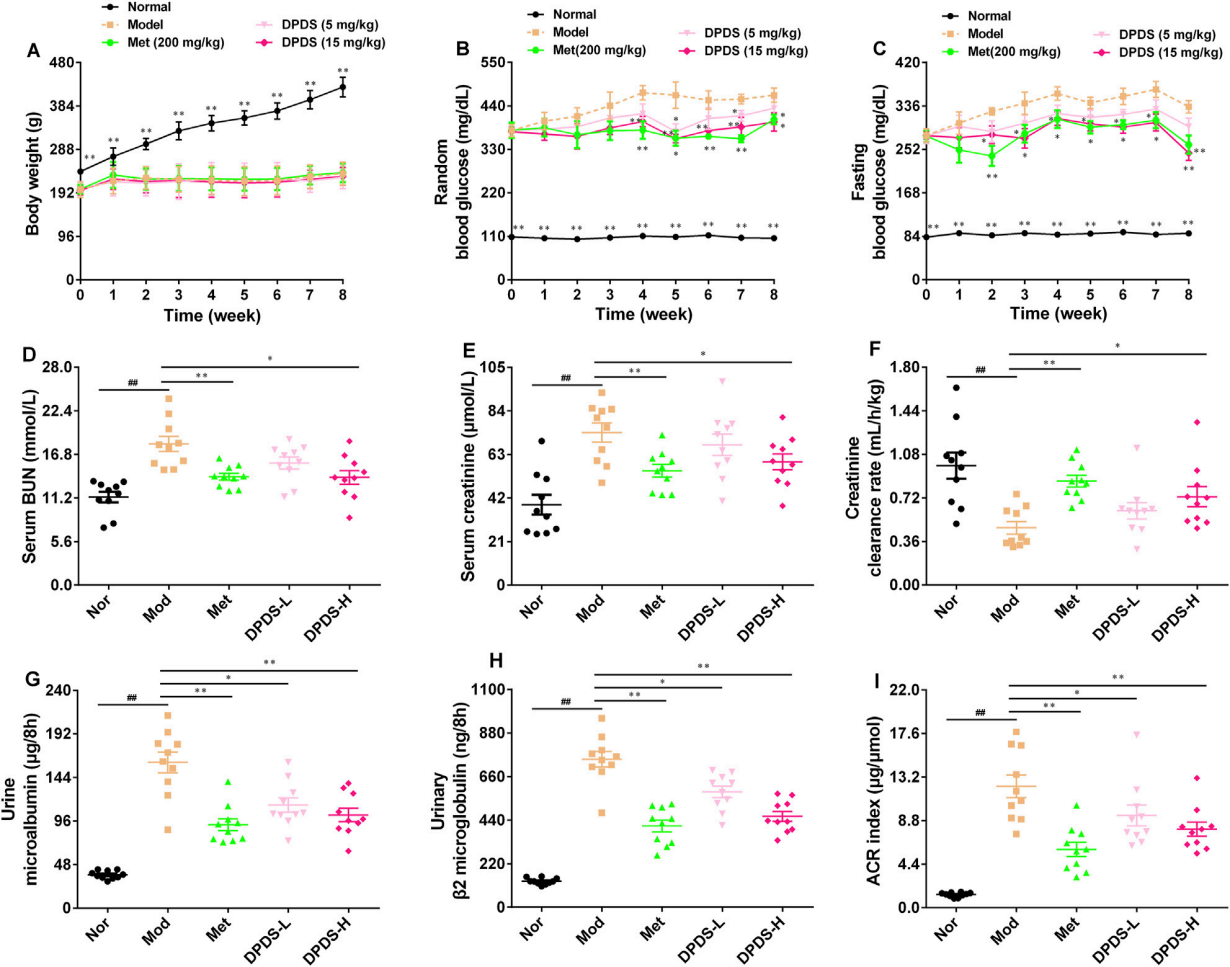
DPDS intervention reduced blood glucose and ameliorated renal dysfunction in DKD rats (*n* = 10, means ± SEM). **(A)** Body weight. **(B)** Random blood glucose concentration. **(C)** Fasting blood glucose concentration. **(D)** Serum BUN. **(E)** Serum creatinine. **(F)** Creatinine clearance rate. **(G)** Urine m-ALB. **(H)** Urine β2-MG. **(I)** ACR index. ^#^ *p* < 0.05; ^##^ *p* < 0.01 vs. Mod group; *p < 0.05; **p < 0.01 vs. Mod group.

During the experiment, the weight and blood glucose of STZ rats were measured every week, and the indexes related to renal function in urine and serum were measured after the experiment. As shown in Figure 1, during the experiment, the weight of rats in the Mod group was significantly lower than that in the Nor group, while the levels of RBG and FBG in this group were markedly higher than those in the Nor group, suggesting the successful establishment of a diabetes model. Relative to the Mod group, high and low doses of DPDS or Met had no effect on the body weight of the STZ rats. However, the high dose of DPDS and Met decreased the RBG level of STZ rats from the fourth week and decreased their FBG level from the second week; the low dose of DPDS had no obvious effect on blood glucose. Moreover, in contrast with the Nor group, the levels of BUN and creatinine in serum as well as the urine m-ALB, urine β2-MG, and american college of radiology (ACR) index were markedly increased, and the Ccr was decreased in the Mod group, suggesting that the kidneys of STZ rats were obviously damaged. Meanwhile, after administration of DPDS or Met for 8 weeks, these abnormal indexes were remarkably restored when compared with the Mod group. In summary, DPDS markedly decreased blood glucose and protected against kidney injury induced by hyperglycemia in STZ rats.

### 3.2 DPDS intervention decreased the lipid content of kidney and attenuated renal pathological changes in DKD rats

**FIGURE 2:**
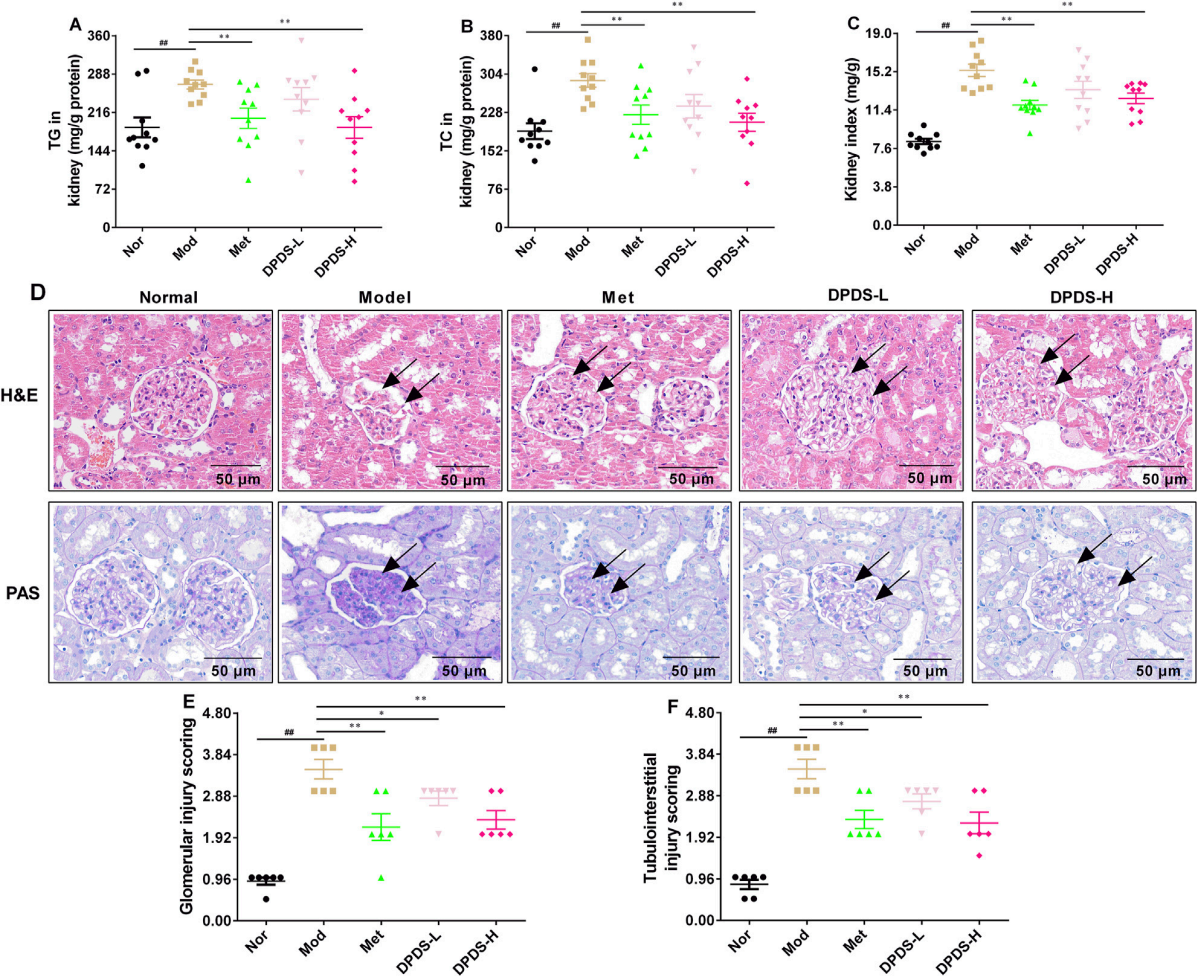
DPDS intervention decreased the lipid content of kidney and attenuated renal pathological changes in DKD rats (means ± SEM). **(A)** TG levels in kidney (*n* = 10). **(B)** TG levels in kidney (*n* = 10). **(C)** Kidney index (*n* = 10). **(D)** Representative pictures of HE and PAS staining (*n* = 4, 200×). **(E)** Glomerular injury score (*n* = 6). **(F)** Tubulointerstitial injury score (*n* = 6). ^#^ *p* < 0.05; ^##^ *p* < 0.01 vs. Mod group; *p < 0.05; **p < 0.01 vs. Mod group.

To investigate the effect of DPDS on lipid content and pathological morphology in the kidney of the STZ rats, the contents of TG and TC were determined, and the kidneys were stained with HE and PAS. As displayed in Figure 2, compared to the Nor group, the contents of TG and TC in the kidney and the renal index were noticeably higher than those in the Nor group. However, after 8 weeks of DPDS treatment, the rats in the DPDS group and Met group exhibited significantly decreased TG and TC levels in the kidney as well as a reduced kidney index compared to the Mod group. Furthermore, HE and PAS staining of the kidneys in the Nor group exhibited normal morphological characteristics, including no inflammatory cell infiltration in the renal area, a complete and clear morphological structure of glomerulus and renal tubules, normal distribution of ECM and mesangial cells, and little glycogen deposition. In contrast with the Nor group, the Mod group’s kidney showed obvious renal structural damage, such as glomerular hypertrophy, glomerular ECM, and basement membrane thickening, more inflammatory cells infiltrating into the renal interstitium, and more glycogen deposition. Intriguingly, after administration of DPDS or Met for 8 weeks, both of the abnormal changes of HE and PAS have been reversed to some extent. Furthermore, the glomerular injury score and tubulointerstitial injury score in Mod group rats was markedly higher than that in Nor group rats, but they were reduced in DPDS treated diabetic rats compared to the Mod group rats. Together with the results of Figure 1, this part showed that DPDS obviously alleviated renal function and protected against renal injury in the STZ-induced DKD rat model.

### 3.3 DPDS intervention alleviated dyslipidemia and relieved systemic oxidative stress in DKD rats

To further confirm the effect of DPDS on serum lipid content and related indexes of oxidative stress in DKD rats, biochemical methods were used to determine the related indexes. As displayed in Table 1, relative to the Nor group, the levels of TG, TC, LDL-C, MDA, and the activities of AST and ALT in the serum were dramatically augmented in the Mod group. Meanwhile, the contents of HDL-C, GSH, along with the activities of T-AOC, SOD, and GSH-Px in the serum were noticeably declined in the Mod group, which indicated that the metabolism of blood lipids was disordered, oxidative stress was increased, and liver function was impaired in DKD rats. However, treatment with a high dose of DPDS or Met was effective against those abnormal changes compared to the Mod group, and the low-dose DPDS reduced the levels of TG, ALT, and AST in the serum, and it had a tendency to improve other indexes. Overall, these results indicated that DPDS improved dyslipidemia in DKD rats and protected them from oxidative stress and liver function damage induced by high glucose.

**TABLE 1:** DPDS intervention alleviated dyslipidemia and relieved systemic oxidative stress in DKD rats.

Name of index	Nor	Mod	Met	DPDS-L	DPDS-H
Blood TG (mg/dL)	112.5 ± 22.5**	338.4 ± 30.6	267.5 ± 36.4**	287.4 ± 43.4*	213.0 ± 38.7**
Blood TC (mg/dL)	108.7 ± 12.7**	168.1 ± 21.3	130.7 ± 19.7**	154.5 ± 32.4	143.9 ± 15.5*
Blood LDL-C (mg/dL)	98.4 ± 12.9**	135.0 ± 9.3	100.7 ± 9.7**	120.2 ± 18.4	116.9 ± 5.3*
Blood HDL-C (mg/dL)	106.5 ± 14.1**	61.0 ± 9.3	78.3 ± 7.6**	68.6 ± 11.7	76.9 ± 10.6**
Blood MDA (nmol/L)	14.4 ± 2.6**	21.0 ± 4.4	15.8 ± 4.0*	20.3 ± 2.9	15.4 ± 3.6*
Blood T-AOC (U/mL)	12.7 ± 3.7**	7.8 ± 1.8	10.8 ± 1.9**	8.8 ± 1.4	10.3 ± 1.9*
Blood SOD (U/mL)	270.5 ± 18.3**	209.4 ± 19.0	247.3 ± 11.8**	223.2 ± 13.2	247.7 ± 18.3**
Blood GSH-Px (U/mL)	46.4 ± 10.8**	30.4 ± 5.4	42.9 ± 15.2*	38.0 ± 14.7	44.2 ± 14.0*
Blood GSH (μmol/L)	128.8 ± 15.8**	99.4 ± 9.5	114.8 ± 11.3*	107.3 ± 6.6	121.5 ± 10.4*
Blood ALT (U/L)	68.4 ± 14.1**	114.5 ± 21.8	80.3 ± 20.6**	93.6 ± 13.8*	77.9 ± 10.4**
Blood AST (U/L)	61.0 ± 12.1**	119.7 ± 16.4	95.3 ± 14.1**	96.9 ± 18.1*	82.7 ± 16.5**

Data are presented as mean ± SEM, *n* = 10. *p < 0.05; **p < 0.01 vs. Mod group. Nor = Normal, Mod = Model, Met = Metformin, DPDS-L, Diphenyl diselenide low-dose; DPDS-H, Diphenyl diselenide high-dose.

### 3.4 DPDS intervention inhibited the production of ECM in kidney of the DKD rats

**FIGURE 3:**
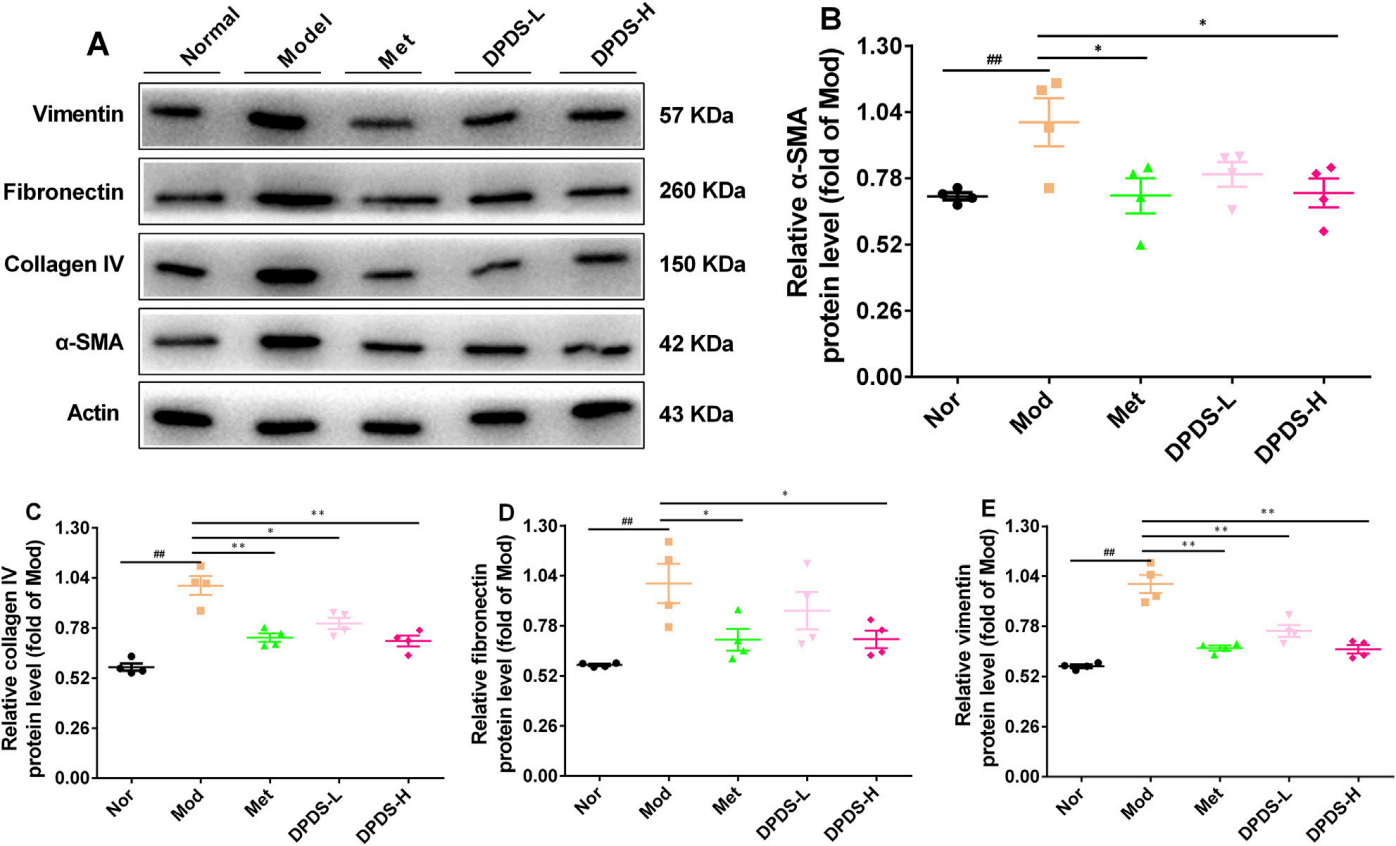
DPDS intervention inhibited the production of ECM in kidney of the DKD rats (*n* = 4, means ± SEM). **(A)** Representative images of Western blot. **(B)** The relative density analysis of α-SMA. **(C)** The relative density analysis of COI IV. **(D)** The relative density analysis of FN. **(E)** The relative density analysis of vimentin. ^#^ *p* < 0.05; ^##^ *p* < 0.01 vs. Mod group; *p < 0.05; **p < 0.01 vs. Mod group.

To further clarify whether DPDS can inhibit the production of ECM in the kidney of DKD rats, the expression of related proteins was detected by Western blot. As shown in Figure 3, in contrast with the Nor group, the Mod group’s kidney showed remarkably elevated protein expressions of α-SMA, COI Ⅳ, FN, and vimentin, suggesting a large amount of ECM was produced in the kidney of DKD rats. However, DPDS reduced the above protein expression in a dose-dependent manner after 8 weeks of intervention. Meanwhile, Met also restored the expression of these proteins. In conclusion, the beneficial effect of DPDS on DKD is closely related to reducing ECM production in the kidney.

### 3.5 DPDS intervention extenuated oxidative stress damage in kidney of the DKD rats

**FIGURE 4:**
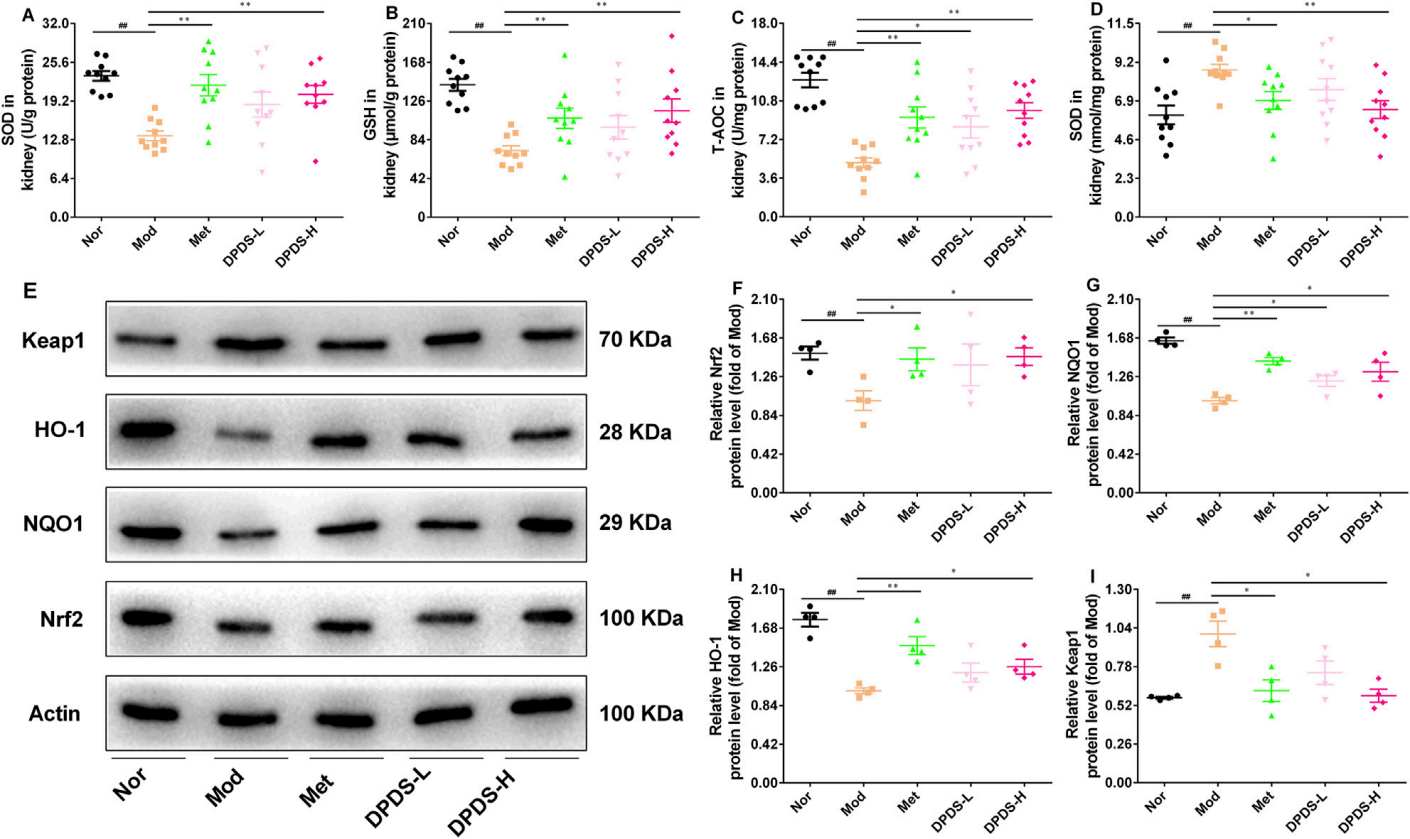
DPDS intervention extenuated oxidative stress damage in kidney of the DKD rats (means ± SEM). **(A)** SOD activities in kidney (*n* = 10). **(B)** GSH levels in kidney (*n* = 10). **(C)** T-AOC activities in kidney (*n* = 10). **(D)** MDA levels in kidney (*n* = 10). **(E)** Representative images of Western blot (*n* = 4). **(F)** The relative density analysis of Nrf2 (*n* = 4). **(G)** The relative density analysis of NQO-1 (*n* = 4). **(H)** The relative density analysis of HO-1 (*n* = 4). **(I)** The relative density analysis of Keap1 (*n* = 4). ^#^ *p* < 0.05; ^##^ *p* < 0.01 vs. Mod group; *p < 0.05; **p < 0.01 vs. Mod group.

To evaluate whether DPDS could protect the kidneys of DKD rats from oxidative stress induced by hyperglycemia, the related biochemical indexes in the kidney were determined. As depicted in Figures 4A–D, compared to the Nor group, the Mod group showed significantly reduced levels of SOD, GSH, and T-AOC, along with markedly elevated MDA content. Nevertheless, DPDS treatment enhanced the activities of these antioxidant enzymes and decreased MDA content in a dose-dependent manner, similar to the effects observed with Met. To further explore the molecular mechanism of DPDS in alleviating renal oxidative stress damage, we used Western blot to detect Nrf2/Keap1 signaling pathway-related proteins. The results shown in Figures 4E–I demonstrated that the protein expressions of Nrf2, NQO1, and HO-1 were lower, and the Keap1 protein levels were higher in the kidneys of the Mod group compared to the Nor group. However, after 8 weeks of treatment with DPDS or Met, the expression levels of these proteins partially recovered, with high-dose DPDS showing more effectiveness. Taken together, these results indicated that DPDS may potentially play a therapeutic role in DKD by reducing renal oxidative stress through regulating Nrf2/Keap1 signaling pathway.

### 3.6 DPDS intervention improved intestinal flora diversity in DKD rat

**FIGURE 5:**
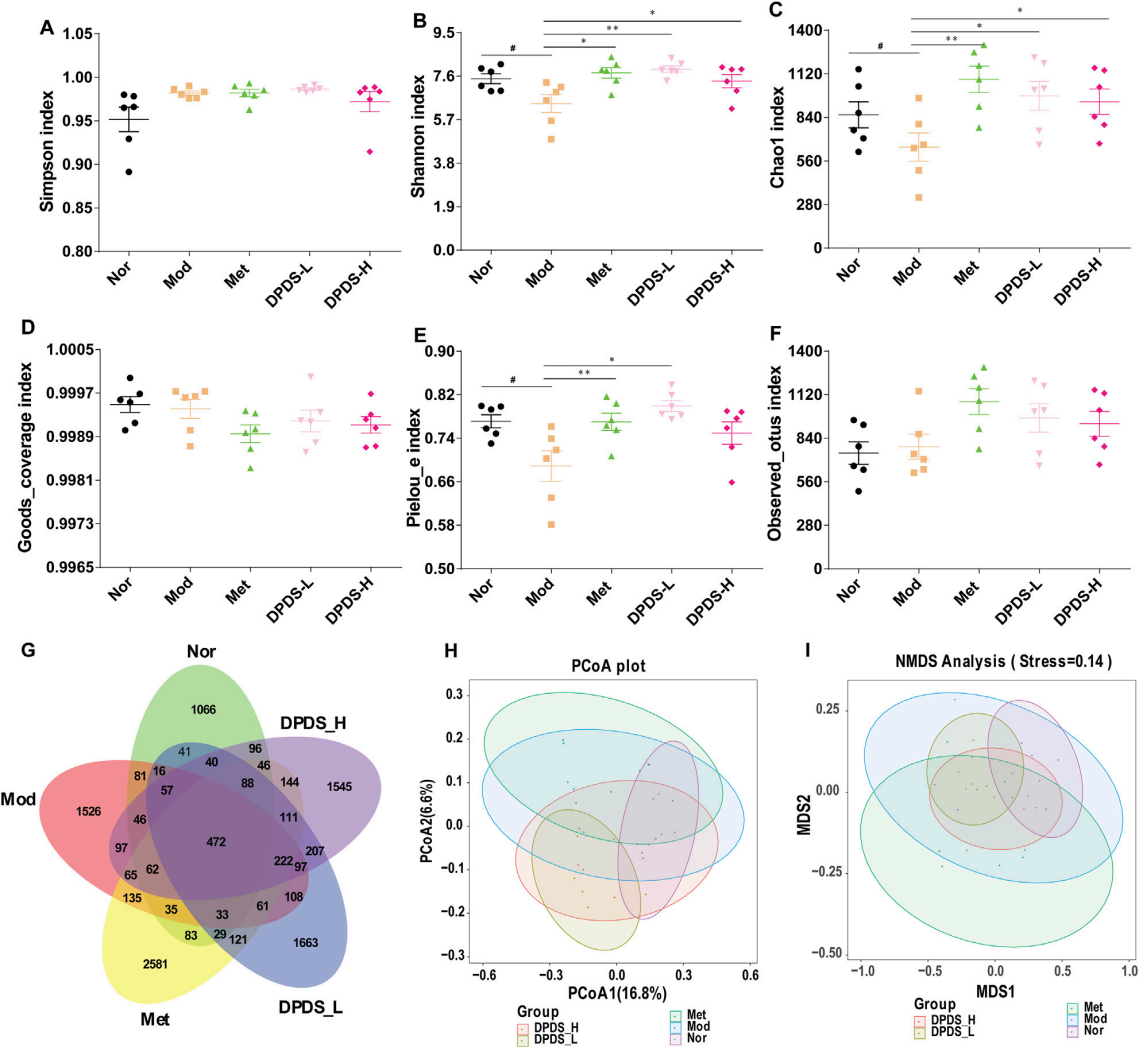
DPDS intervention ameliorated intestinal flora diversity in DKD rats (*n* = 6, means ± SEM). **(A)** Simpson index. **(B)** Shannon index. **(C)** Chao1 index. **(D)** Goods_coverage index. **(E)** Pielou-e index. **(F)** Observed_otus index. **(G)** Venn diagram. **(H)** PCoA analysis diagram. **(I)** NMDS analysis diagram. ^#^ *p* < 0.05; ^##^ *p* < 0.01 vs. Mod group; *p < 0.05; **p < 0.01 vs. Mod group.

To further clarify the relationship between the anti- DKD effect of DPDS and the intestinal microflora, the microflora in the feces of rats in each group was analyzed. As illustrated in Figures 5A–F, regarding α diversity, no significant differences were observed in the three indexes (Simpson index, Goods_coverage index, and Observed_otus index) among the five groups. However, for the main parameters of α diversity, the Mod group exhibited lower Shannon index, Chao1 index, and Pielou-e index in comparison with Nor group. Nevertheless, supplementation with DPDS noticeably increased these three indexes in comparison with Mod group. The Venn diagram in Figure 5G showed that there are 2291, 3113, 4288, 3366 and 3395 OTUs in theNor, Mod, Met, DPD-L and DPD-H groups, respectively; and the number of reciprocal OTUs shared across the different groups was 472. Furthermore, β diversity was used to evaluate the community structure of the samples. As illustrated in Figures 5H, I, the results of PCoA and NMDS analysis in the Mod group and the Nor group are obviously different. However, after 8 weeks of treatment with DPDS and Met, the community structure in these groups became closer to that of the Nor group. In summary, these results indicated that DPDS could increase the richness and diversity of the microbial community and restore the abnormal community structure in DKD rats.

### 3.7 DPDS intervention improved the composition of intestinal flora in DKD rat

**FIGURE 6:**
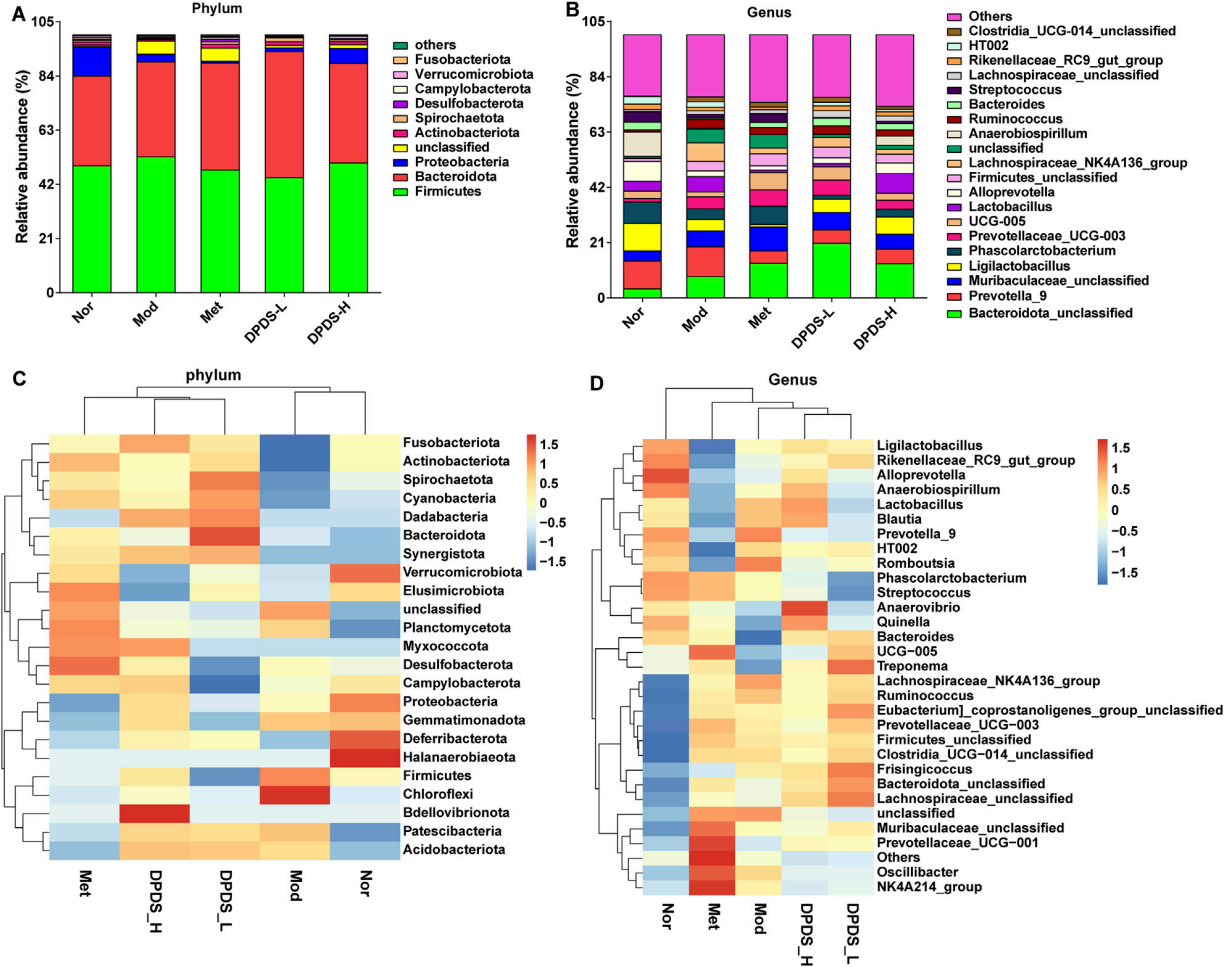
DPDS intervention improved the composition of intestinal flora in DKD rat (*n* = 6, means ± SEM). **(A)** Phylum level species distribution. **(B)** Genus level species distribution. **(C)** Heat map of species distribution at the phylum level. **(D)** Heat map of species distribution at the genus level.

**FIGURE 7:**
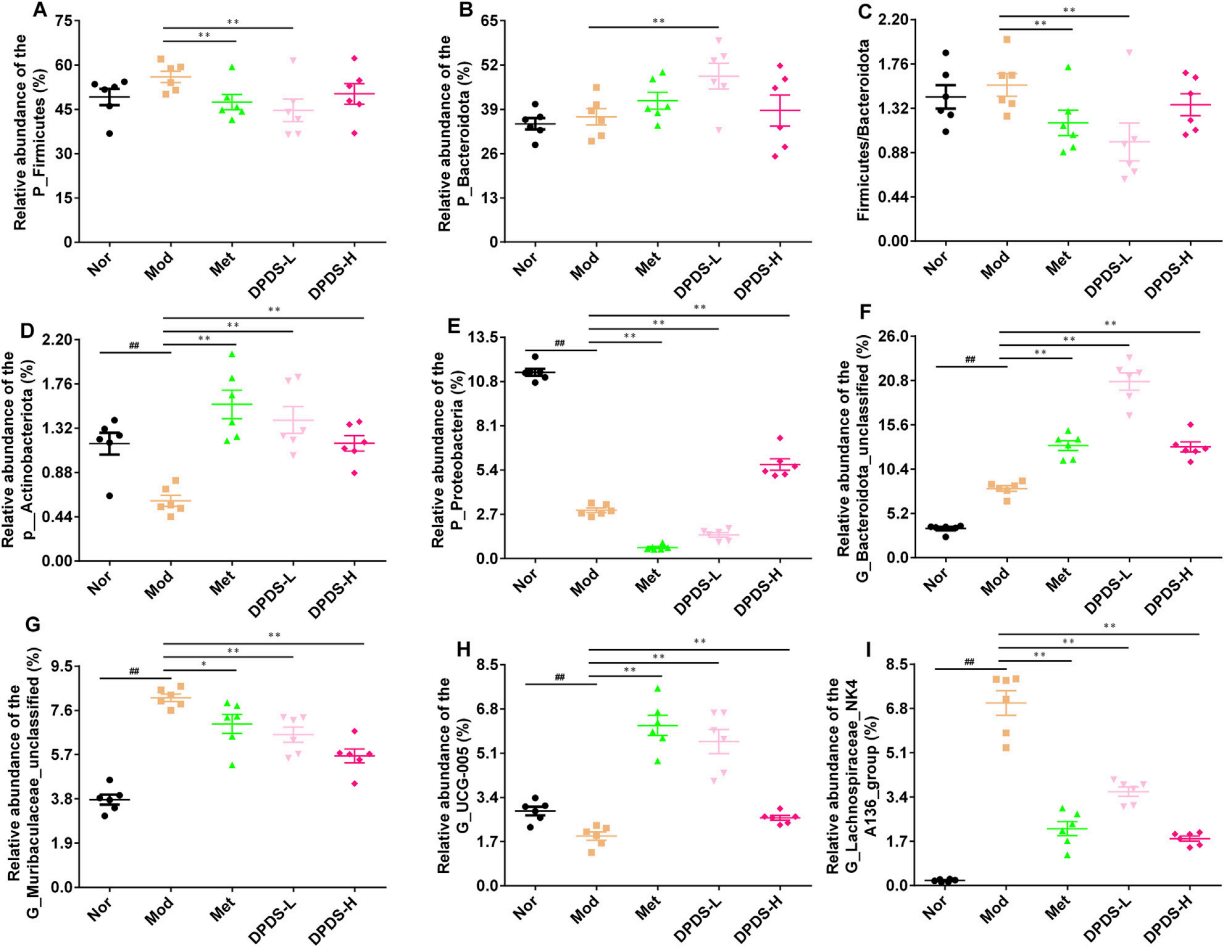
DPDS intervention ameliorated the intestinal flora composition at levels of phylum and genus in DKD rats (*n* = 6, means ± SEM). **(A)** P_Firmicutes. **(B)** P_Bacteroidota. **(C)** The ratio of Firmicutes/Bacteroidota. **(D)** P_Actinobacteriota. **(E)** P_Proteobacteria. **(F)** G_Bacteroidota_unclassified. **(G)** G_Muribaculaceae_unclassified. **(H)** G_UCG-005. **(I)** G_Lachnospiraceae_NK4A136_group. ^#^ *p* < 0.05; ^##^ *p* < 0.01 vs. Mod group; *p < 0.05; **p < 0.01 vs. Mod group.

We further analyzed the composition of intestinal microorganisms in each group at the phylum and genus levels. The top 10 phyla and top 20 genera of intestinal flora in each group are displayed in Figures 6A, B. At the phylum level, Firmicutes, Bacteroidetes, Proteobacteria, as well as Unclassified accounted for more than 90% of the total microorganisms and were the dominant phyla. At the genus level, although the dominant genera were not concentrated, the composition of each group was clearly different. The results of phylum and genus species distribution heatmap in Figures 6C, D further showed that the microbial composition of each group was obviously different, consistent with the results of the columnar stacking diagram. Further statistical analysis of dominant strains at the phylum and genus levels, as indicated in Figures 7A–I, showed that in comparison to the Nor group, the Mod group exhibited increased relative abundance of Firmicutes, Bacteroidota and the Firmicutes/Bacteroidota ratio, along with a decreased relative abundance of Actinobacteriota and Proteobacteria. At the genus level, in contrast with the Nor group, the Mod group increased relative abundance of Bacteroidota_unclassified, Muribaculaceae_unclassified, and Lachnospiraceae_NK4A136_group, and a decreased relative abundance of UCG-005. After DPDS intervention for 8 weeks, the composition of bacteria at both the phylum and genus levels tended towards that of the normal group. Overall, these findings revealed that DPDS could restore the composition and diversity of intestinal flora in DKD rats.

### 3.8 DPDS intervention altered the abundances of different taxa in each group of DKD rats

**FIGURE 8:**
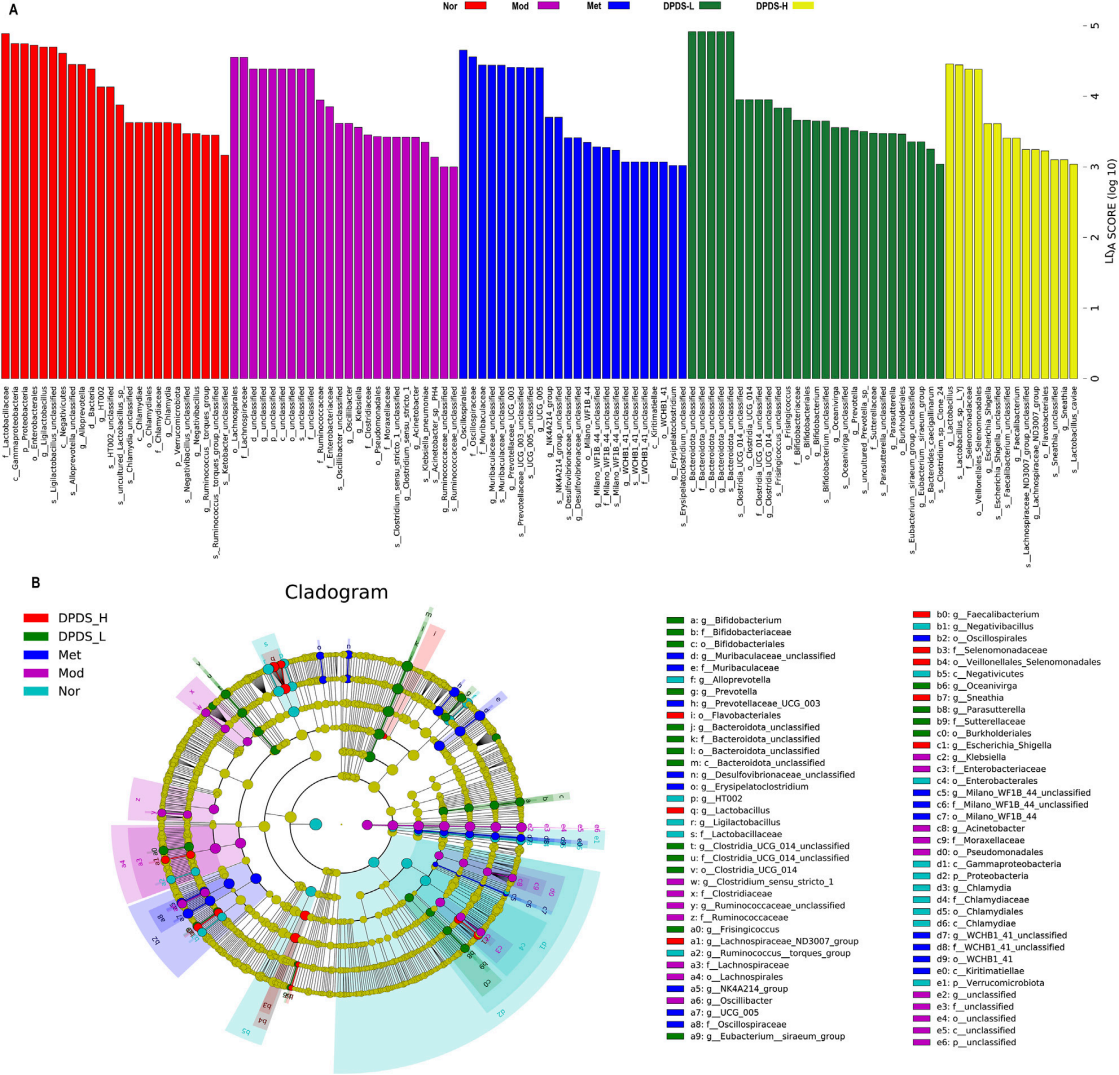
DPDS intervention changed the differentially abundant taxa of each group in DKD rats (*n* = 6, means ± SEM). **(A)** Histogram of LDA values (lg 10 > 3). **(B)** Taxonomic cladogram obtained by LEfSe.

Next, we utilized the linear discriminant analysis effect size (LEfSe) and linear discriminant analysis (LDA) to examine the differences among the various groups and identify enriched taxonomic groups. Based on the criterion (LDA score >3), the results displayed in Figures 8A, B revealed a total of 113 different OTUs in the five groups. There were 24 biomarkers in Nor groups, primarily at the genus level (Ligilactobacillus, Alloprevotella, HT002, *Chlamydia*, Negativibacillus, Ruminococcus_torques_group), species level (Ligilactobacillus, Alloprevotella, HT002_unclassified, uncultured_*Lactobacillus*_sp, *Chlamydia*_unclassified, Negativibacillus_unclassified, Ruminococcaceae_unclassified), and several other genera. There were 24 biomarkers in Mod groups, mainly at the family level (Lachnospiraceae, Ruminococcaceae, Clostridiaceae), genus levels (Oscillibacter, *Clostridium*_sensu_stricto_1, *Acinetobacter*), species levels (Oscillospira_unclassified, *Klebsiella*_pneumoniae, *Clostridium*_sensu_stricto_1_unclassified) and several other genera. There were 24 biomarkers in DPDS-L groups, primarily at the genus level (Muribaculaceae_unclassified, Prevotellaceae_UCG-003, NK4A214_group), species levels (UCG-005_unclassified, Prevotellaceae_UCG-003_unclassified, Desulfovibrionaceae_unclassified) and several other genera. There were 27 biomarkers in DPDS-L groups, with most at the genus level (Bacteroidota_unclassified, Frisingicoccus, Bifidobacterium), species level (Clostridia_UCG-014_unclassified, Frisingicoccus_unclassified, Bifidobacterium_unclassified) and several other genera. There were 14 biomarkers in DPDS-H groups, mainly at the genus level (*Lactobacillus*, Escherichia-Shigella, Faecalibacterium), species level (*Lactobacillus*_sp._L-YJ, Escherichia-Shigella_unclassified, Sneathia_unclassified) and several other genera. Overall, these findings further indicated that DPDS influenced the composition of the intestinal microflora in DKD rats.

### 3.9 Predictive results of bacterial phenotype

**FIGURE 9:**
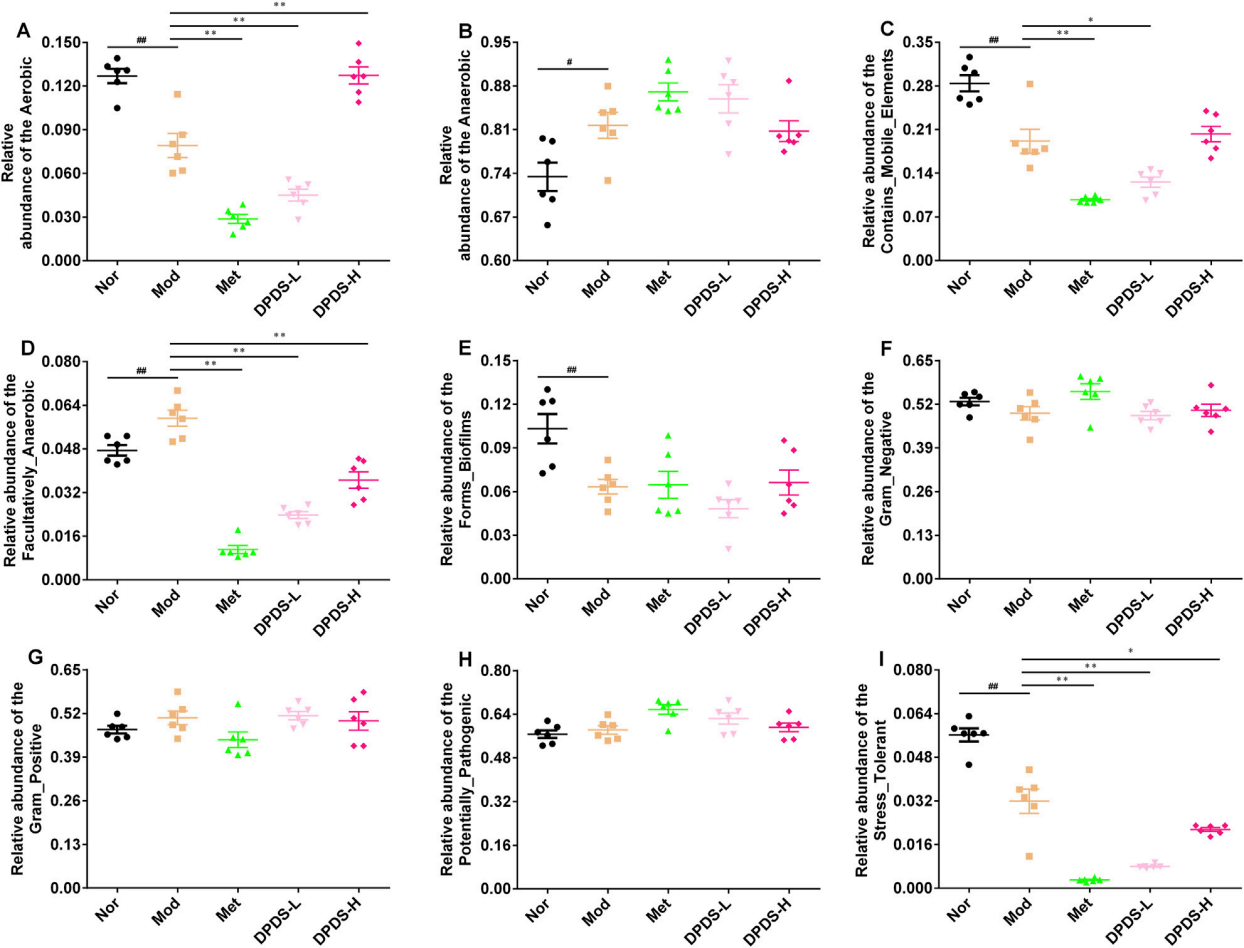
Predictive results of bacterial phenotype (*n* = 6, means ± SEM). **(A)** The relative abundance of Aerobic. **(B)** The relative abundance of Anaerobic. **(C)** The relative abundance of Contains Mobile Elements. **(D)** The relative abundance of Facultatively Anaerobic. **(E)** The relative abundance of Forms Biofilms. **(F)** The relative abundance of Gram-Negative. **(G)** The relative abundance of Gram-Positive. **(H)** The relative abundance of Potentially Pathogenic. **(I)** The relative abundance of Stress Tolerant. ^#^ *p* < 0.05; ^##^ *p* < 0.01 vs. Mod group; *p < 0.05; **p < 0.01 vs. Mod group.

Finally, we used BugBase to predict 9 potential bacterial phenotypes in each group. The findings are illustrated in Figures 9A–I. Compared to the Mod group, both DPDS and Met had obvious effects on the following species: Aerobic, Contains Mobile Elements, Facultatively Anaerobic, and Stress Tolerant. However, they did not have a noticeable impact on Anaerobic, Forms Biofilms, Gram-Negative, Gram-Positive, and Potentially Pathogenic phenotypes.

### 3.10 Correlation between the intestinal microbiota and the related indexes of DKD

**FIGURE 10:**
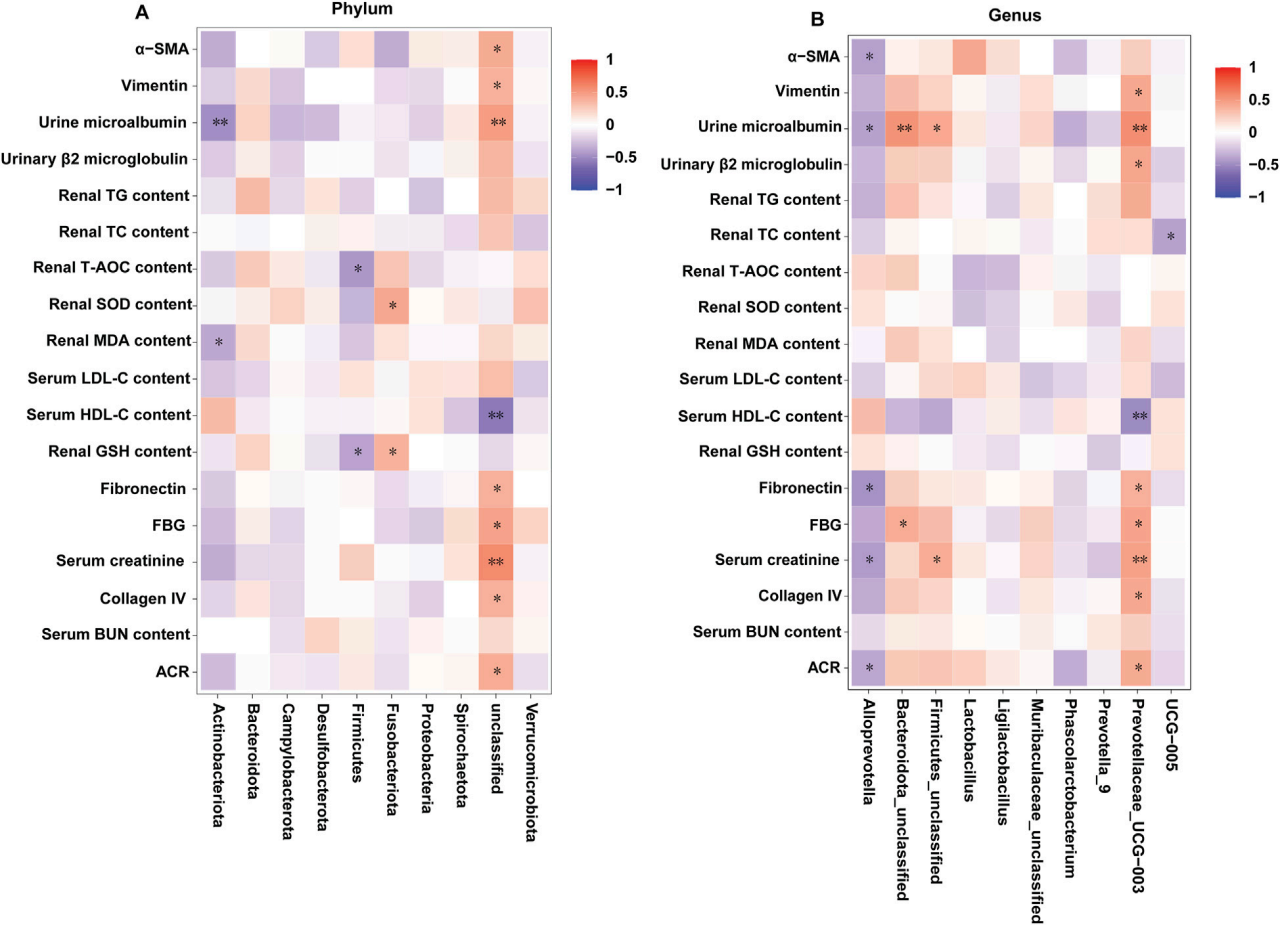
Correlation between the intestinal microbiota and diarrhea-related index (*n* = 6, means ± SEM). **(A)** Spearman correlation analysis between intestinal flora and the related indexes of DKD at the phylum level. **(B)** Spearman correlation analysis between intestinal flora and the related indexes of DKD at the genus level.

The correlations between the related indexes of DKD and the gut microbes in all the groups were investigated by the spearman’s correlation analysis. As depicted in Figures 10A, B, at the phylum level, the urine microalbumin and renal MDA content were markedly and negatively associated with Actinobacteriota; the levels of T-AOC and GHS in kidney were markedly and negatively associated with Firmicutes; the levels of SOD and GHS in kidney were positively associated with Fusobacteriota; the levels of α-SMA, Vimentin, m-ALB, FN, FBG, serum creatinine, collagen IV and ACR index were obviously and positively associated with unclassified, and the serum HDL−C levels was negatively associated with unclassified. At the genus level, the levels of α-SMA, m-ALB, FN, serum creatinine and ACR index were negatively associated with Alloprevotella; the levels of m-ALB and FBG were positively associated with Bacteroidota-unclassified; the levels of m-ALB and serum creatinine were positively associated with Firmicutes-unclassified; the levels of Vimentin, m-ALB, β2-MG, FN, FBG, serum creatinine, collagen IV and ACR index were obviously and positively associated with Prevotellaceae-UCG-003, and the serum HDL−C levels was negatively associated with Prevotellaceae-UCG-003; the levels of TC in kidney was negatively associated with UCG−005. Overall, the identified bacteria are closely related to the indicators for evaluating DKD.

## 4 Discussion

DKD is one of the main causes of death in diabetic patients. Dialysis or organ transplantation is the only treatment option for DKD in the advanced stages, but they can significantly compromise the health of individuals. Unfortunately, there are limited drugs available to effectively treat DKD at present. Thus, it is urgent to develop novel medications to slow down the progression of DKD. While our previous research had demonstrated the ability of DPDS to improve type 1 DKD by reducing oxidative stress and inflammatory reactions, it remains uncertain whether DPDS can improve renal ECM and intestinal flora in type 1 diabetic rats. In this study, we studied the protective effect and potential mechanism of DPDS on DKD in STZ-induced type 1 diabetic rats. Based on the results obtained from biochemical index determination, HE staining, and PAS staining, DPDS significantly improved the indexes related to DKD lesions and alleviated renal histopathological abnormalities, consistent with our previous studies (Wang et al., 2021). Moreover, the findings also showed that DPDS inhibited the production of renal ECM, alleviated the oxidative stress injury of kidney, and regulated intestinal microbial composition. These outcomes provided valuable data support for further development and application of DPDS in the context of DKD.

Sustained hyperglycemia is the initial trigger for the progression of DKD, and controlling blood gluocose levels represents one of the primary strategies to treat DKD (Mohandes et al., 2023). Additionally, persistent hyperglycemia will affect the growth and metabolism of intestinal bacteria, leading to intestinal bacteria imbalance, leading to the decrease of beneficial bacteria and the increase of conditional pathogenic bacteria, which will lead to intestinal inflammation and infection (Gu et al., 2024). Moreover, persistent hyperglycemia will damage the intestinal mucosal barrier, make harmful substances and bacteria in the intestine enter the blood circulation, destroy the composition and diversity of intestinal flora, and trigger systemic inflammatory response (Abuqwider et al., 2023). Diabetic patients often experience lipid metabolism disorders, and excessive lipid accumulation can result in lipotoxicity, leading to cellular damage and organelle dysfunction (Zhu et al., 2023). Furthermore, excessive lipid accumulation in the kidneys affects both glomerular filtration rate and proteinuria. Additionally, it promotes the generation of ROS, thereby facilitating the development of DKD (Zhu et al., 2023; Opazo-Ríos et al., 2020). In this study, supplementation with DPDS demonstrated a partial reduction in RBG and FBG levels. Moreover, it decreased the levels of TG and TC in both serum and kidney tissues of DKD rats, aligning with our observations and previous studies (Wang et al., 2021; Dos Santos et al., 2020; da Rocha et al., 2011). These results indicate that DPDS can mitigate renal damage caused by hyperglycemia and lipotoxicity in type 1 diabetic rats. Although the hypoglycemic effect of DPDS might not be significant, it partially elucidates the protective impact of DPDS on DKD progression.

One of the key pathological characteristics of kidney disease in DKD rats is renal interstitial fibrosis, primarily characterized by the deposition of ECM (Li and Ma, 2023). Normally, the generation and degradation of renal ECM are in a dynamic balance, maintaining the normal function of the kidney (Hansen et al., 2015). However, in cases of DKD, persistent hyperglycemia promotes the production of fibrinogen and FN. Additionally, inflammatory cells infiltrating the kidneys release soluble cytokines, growth factors, and vasoactive substances, facilitating the penetration of plasma proteins (including fibrinogen and FN) into damaged sites, resulting in ECM deposition (Han et al., 2021; Hansen et al., 2015). Appropriate ECM can bind fibroblasts and immune cells to damaged areas, aiding in the repair or removal of pathogens. However, if the damage is severe or if excessive and persistent ECM production occurs, the ECM will break down into active fragments, activating surrounding cells to produce more difficult-to-degrade fibrotic molecules such as COI and fibrin. Consequently, an increasing amount of COI and FN accumulates in glomeruli and renal tubules, forming a dense and sturdy ECM, which leads to changes in microenvironment of affected areas, directly impacting renal function and pathological changes in the kidneys (Tung et al., 2018). The main components of renal ECM include α-SMA, COI Ⅳ, FN, and vimentin. α-SMA, a member of the actin family, plays a crucial role in the renal tissue injury of DKD. It participates in the synthesis of FN, type Ⅰ COI, type Ⅲ COI, and other ECM components. Studies have indicated that the increased expression of α-SMA in renal tissue of patients with DKD correlates with the severity of renal tissue injury and can also influence the apoptosis process of fibroblasts, renal tubular epithelial cells, and other cells. Consistent with the results of previous studies, the kidneys of STZ-induced diabetic rats exhibited remarkably elevated protein expressions of α-SMA, COI Ⅳ, FN, and vimentin. However, treatment with DPDS noticeably reduced these protein expressions. These results indicate that the decrease of renal ECM in diabetic rats is related to the improvement of kidney function through DPDS treatment.

Among many potential mechanisms leading to the development of DKD, oxidative stress has been proven to play an important role in its occurrence and progression (Jin et al., 2023). The kidney, compared to other organs, is extremely sensitive to oxidative stress (Geng et al., 2018). Damage to the kidney is primarily caused by sustained hyperglycemia, which induces more oxygen free radicals. This damage includes several aspects. Firstly, persistent hyperglycemia can lead to an excess of ROS, which can cause glomerular mesangium, endothelial cells, and podocytes to undergo apoptosis and detach from the basement membrane, thus destroying the integrity of the glomerular filtration membrane and even leading to proteinuria (Ma et al., 2023). Secondly, excessive ROS can interact with biomolecules such as lipids, proteins, and DNA. This interaction can damage the structure of these molecules and activate a series of cell signal pathways, potentially resulting in severe renal dysfunction or injury (Darenskaya et al., 2023). For instance, ROS can directly activate the protein kinase C (PKC) pathway, mitogen can activate protein kinase (MAPK) pathway, and nuclear factor-κB (NF-κB) signaling pathway, thus promoting the advancement of DKD (Ma et al., 2023). Lastly, a high concentration of glucose can promote mesangial cells to increase ECM production in several ways: (1) ROS can promote the expression of plasminogen activator inhibitor, reducing the degradation of fibrin and resulting in more ECM accumulation in the kidney (Qiao et al., 2019); (2) ROS can enhance transforming growth factor-β (TGF-β) expression, which plays a crucial role in regulating the production of renal ECM by activating the PKC and NF-κB signal pathways and promoting ECM accumulation (Qiu et al., 2023); (3) the renin angiotensin converting system and TGF-β/Smad signaling pathway are closely related to the accumulation of renal ECM, and ROS can promote the production of ECM by activating these (Zheng et al., 2011). Reducing ROS production or eliminating excessive ROS is an effective strategy to alleviate the progression of DKD. To further clarify whether DPDS can improve ECM accumulation in the kidneys of DKD rats by reducing oxidative stress, relevant indices in both the serum and the kidney were determined. The results showed that DPDS supplementation notably enhanced the activity of antioxidant enzymes, increased the content of antioxidant substances, but decreased the content of MDA in the kidney and serum of DKD rats. It is necessary to further explore the possible molecular mechanism of DPDS, thereby improving oxidative stress. Nrf2 is the key transcription factor for endogenous resistance to oxidative stress in cells. It regulates the transcription and expression of over 200 genes and is of great significance in the prevention and treatment of DKD. Under oxidative stress, Nrf2 enters the nucleus and combines with the antioxidant response elements, activating antioxidant genes including HO-1, NQO1, and GCLC, which then counteract ROS (Xu et al., 2019). In this study, DPDS administration increased the protein levels of Nrf2, NQO1, HO-1, but decreased the protein expression of Keap1, in agreement with our previous observations and those of others (Wang et al., 2021; Wang et al., 2023; Müller et al., 2018; Mancini et al., 2014). Based on these results, it can be speculated that DPDS may reduce the oxidative stress in the kidneys of DKD rats by regulating the Nrf2/Keap1 signaling pathway. This, in turn, improves the accumulation of ECM in the kidney, playing a protective role against DKD. Although our current and past research results have shown that DPDS can activate the Nrf2 signaling pathway, it is not yet clear whether Nrf2 is the key target for DPDS in preventing and treating DKD. Thus, further studies are necessary to clarify the specific interpretations of these results.

The ecological imbalance of the intestinal microflora is the direct cause of inflammatory bowel disease, obesity, cardiovascular metabolic diseases, diabetes, and related diabetic complications (Crudele et al., 2023). More and more studies show that changes in the intestinal microflora may be important for the occurrence and development of DKD. However, the specific mechanism has not been clarified, and therapy for the intestinal microflora is considered a new strategy to prevent and treat DKD (Han Y. Z et al., 2023). In order to explore whether the effect of DPDS on improving DKD is related to the intestinal flora, the diversity of the intestinal flora in the feces of rats in each group was first investigated. α diversity refers to the diversity in a specific environment or ecosystem, mainly used to reflect species richness, uniformity, and sequencing depth. β diversity refers to species differences between different environmental communities. Changes in the species richness, diversity, or composition of the intestinal microflora may have a profound impact on host physiology by affecting nutrient utilization and the synthesis of bioactive metabolites (Das and Gnanasambandan, 2023). In the present study, DPDS improved the α and β diversity of DKD rats, making them tend towards the Nor group. The microbial communities in the feces of each group were then analyzed. At the phylum level, Bacteroidetes and Firmicutes are the main components of the microbial community (Su et al., 2022). The decrease in *Bacteroides* and the increase in Firmicutes may be related to the existence of genes encoding enzymes that decompose polysaccharides (Li et al., 2018). The F/B ratio is often regarded as a sign of obesity and related to glucose levels. Additionally, the F/B ratio is crucial in reflecting the influence of the intestinal flora on health, and an increase in the ratio usually indicates an unhealthy physiological state (Ma et al., 2022). Here, DPDS supplementation tended to decrease the abundance of Firmicutes and the F/B ratio while increasing the abundance of *Bacteroides* in feces. Moreover, DPDS supplementation also affected other communities at the phylum and genus levels, such as Muribaculaceae_unclassified, Lachnospiraceae-NK4A136-group, UCG-005, and Lachnospiraceae-NK4A136-group. Based on these results, it can be observed that DPDS might have an effect on the intestinal microbial community in the feces of DKD rats. However, the effect is not significant, the possible explanation for this difference is that the composition and diversity of the intestinal microbiota form a complicated system affected by various genetic/environmental factors, including species, age, sex, stress, and diet. It may even change in different disease states or stages within the same individual. Therefore, the detailed mechanism of DPDS improving DKD by affecting intestinal microbial function needs to be revealed in future research. Likewise, to further reveal the relationship between intestinal flora and DKD, we conducted a correlation analysis on the data at the phylum and genus level. The correlation analysis also showed negative correlations of Actinobacteriota, Firmicutes, unclassified, Alloprevotella and UCG-005 with some of renal function and fibrosis related indexes (urine microalbumin, α-SMA, FN, serum creatinine, ACR index), oxidative stress and lipid metabolism factors (MDA, T-AOC, GSH and TC in kidney). Correlation analysis also showed positive correlations of Fusobacteriota, unclassified, Bacteroidota_unclassified, Firmicutes_unclassified and Prevotellaceae-UCG-003 with renal function and fibrosis related indexes (urine microalbumin, α-SMA, vimentin, COI Ⅳ, FN, serum creatinine, β2-MG, ACR index), oxidative stress factor (SOD, GSH in kidney) and the physiological index (FBG). These data revealed that DPDS does have an effect on the intestinal flora of DKD rats, but it is still necessary to use fecal transplantation to prove whether DPDS could improve the renal fibrosis of DKD by regulating these different intestinal microfloras. Furthermore, the results of bacterial phenotype prediction using BugBase indicated that DPDS might have an obvious influence on Aerobic, Contains Mobile Elements, Facultatively Anaerobic, and Stress Tolerant, providing some guidance for our subsequent related research.

However, our research had several limitations. Firstly, Immunofluorescence or immunohistochemistry were not used to identify the specific location of DPDS in kidney, and Sirius Red or Masson Trichrome staining were not used to confirm the role of DPDS in renal fibrosis, which reduced the persuasiveness of our research. Secondly, in this study, we only discussed the effect of DPDS on the composition of fecal intestinal flora in diabetic rats, and did not analyze the specific metabolites (such as short-chain fatty acids and bile acids). It would be more convincing and more meaningful if supported by these relevant data. Finally, Finally, our research was carried out in rats, and its molecular mechanism was not studied *in vitro*. Therefore, further investigation is needed to explore the protective mechanism of DPDS for DN and provide a more robust and comprehensive understanding of DPDS’s pharmacological action.

**FIGURE 11:**
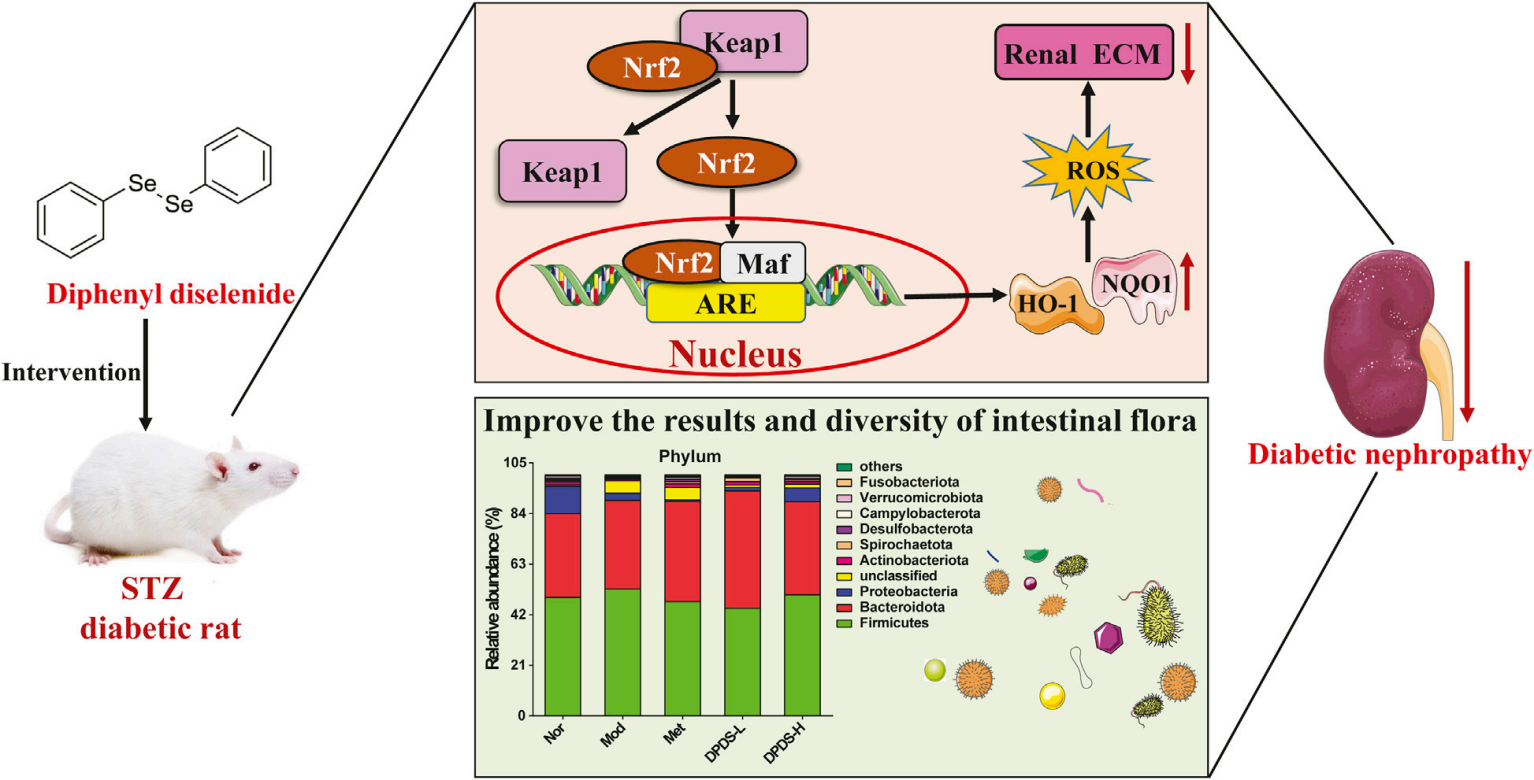
A proposed schematic diagram for the protective mechanisms of DPDS against diabetic nephropathy.

In conclusion, our research revealed that DPDS might have the ability to improve renal dysfunction of STZ-induced diabetic rats. The mechanism might involve regulating the Nrf2/Keap1 signaling pathway to alleviate renal oxidative stress injury, improve intestinal microflora diversity, stabilize intestinal microflora, ameliorate renal ECM deposition, and finally alleviate renal injury in DKD rats (Figure 11). These results indicate that DPDS may be a potential therapeutic drug for DKD, providing new insights into the potential renal protective mechanisms of DPDS.

## Data Availability

The original contributions presented in the study are publicly available. The BioProject accession is PRJNA1190581, and the data can be found here: https://www.ncbi.nlm.nih.gov/sra/PRJNA1190581.
