# Supplementary material for: Diphenyl diselenide protects against diabetic kidney disease through modulating gut microbiota dysbiosis in streptozotocin-induced diabetic rats
Source: Front Pharmacol. 2024 Dec 3;15:1506398. doi: 10.3389/fphar.2024.1506398 (PMC11653185; doi:10.3389/fphar.2024.1506398)
Supplement: Supplementary file 1 [file DataSheet1.docx]

1. Original western blot pictures of the first blots in figure3, the sequence of band from left to right is Normal group, Model group, Metformin group, DPDS-L group and DPDS-H group.


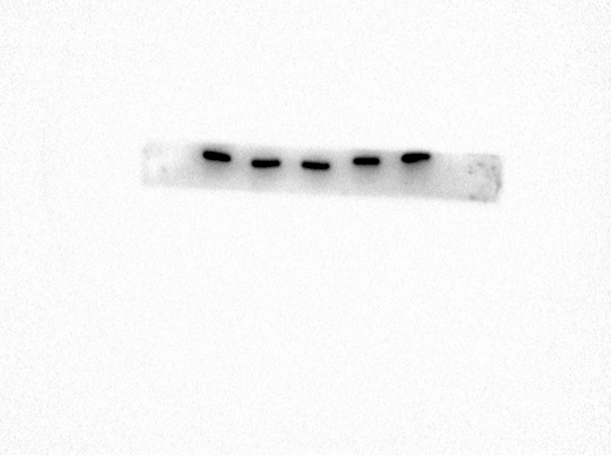

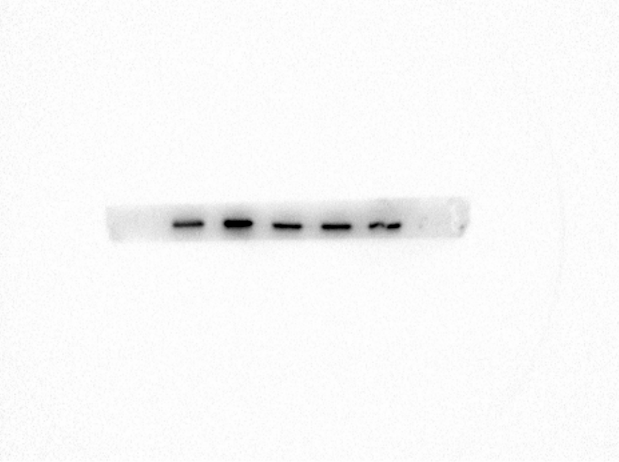


Figure 1: Actin. Figure 2: α-SMA.


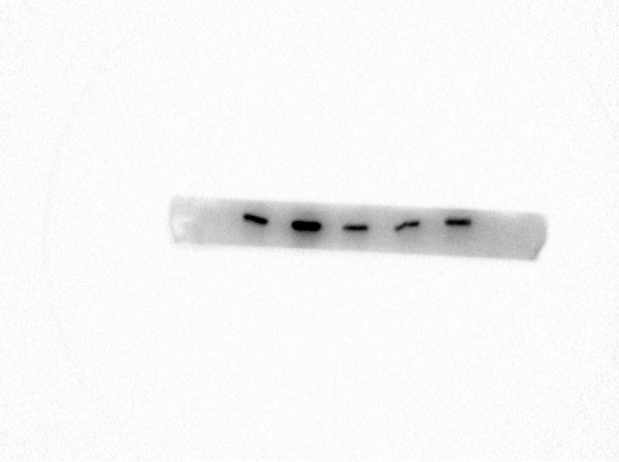

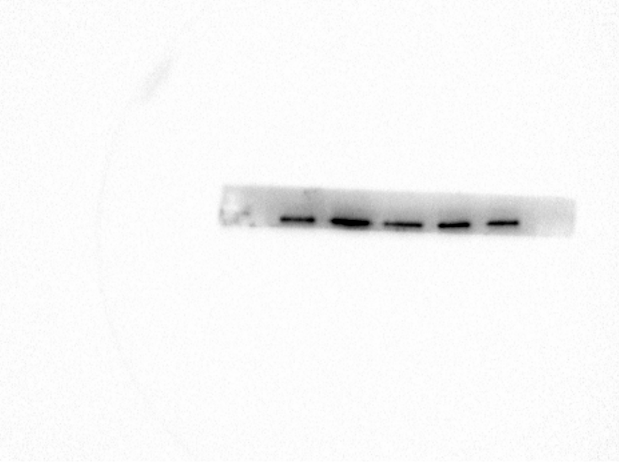


Figure 3: Collagen IV. Figure 4: Fibronectin.


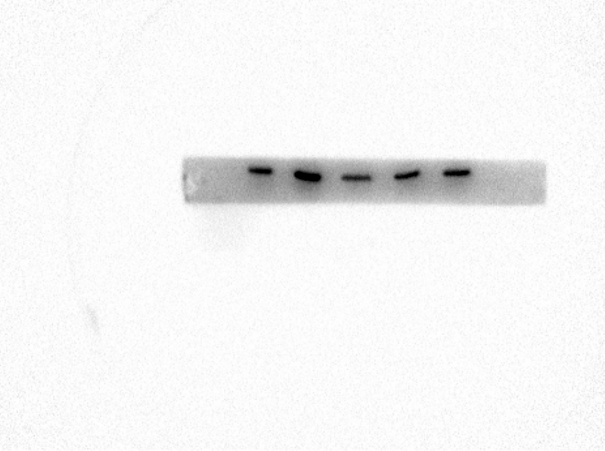


Figure 5: Vimentin.

2. Original western blot pictures of the second blots in figure3, the sequence of band from left to right is Normal group, Model group, Metformin group, DPDS-L group and DPDS-H group.


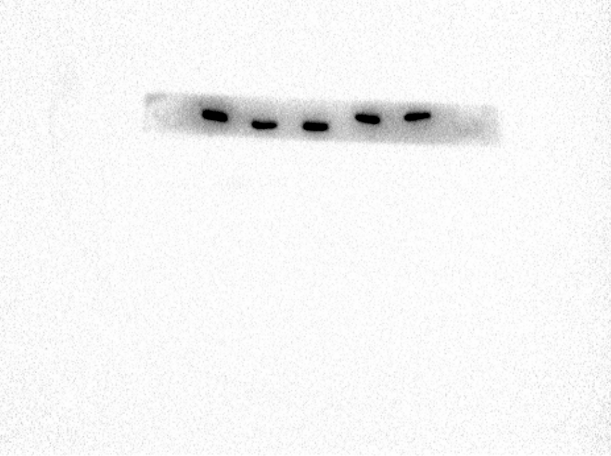

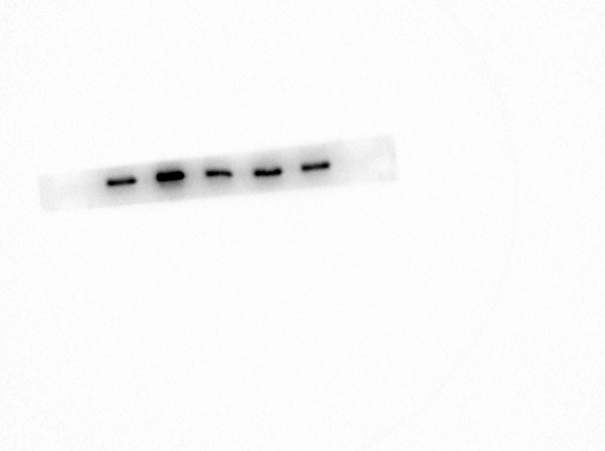


Figure 1: Actin. Figure 2: α-SMA.


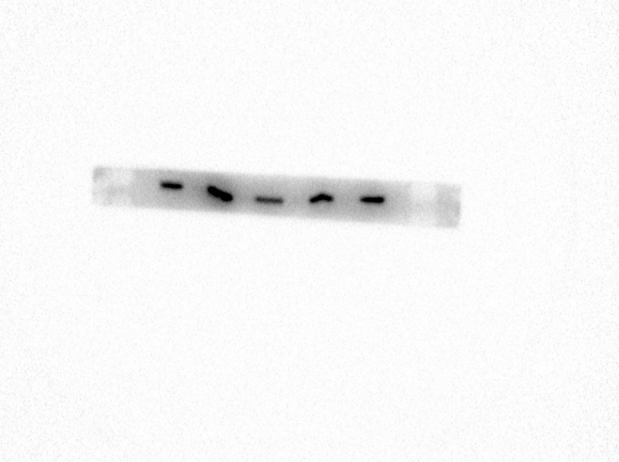

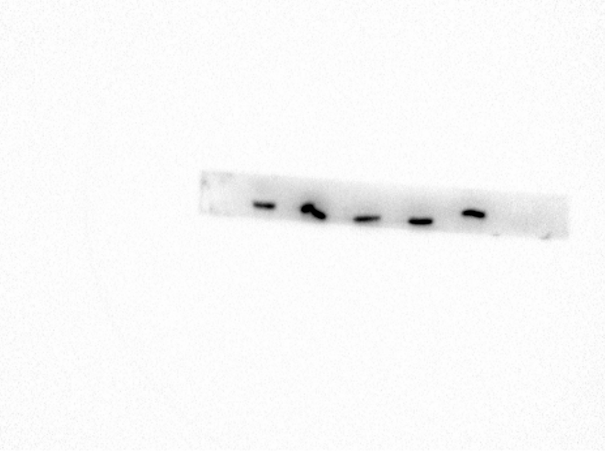


Figure 3: Collagen IV. Figure 4: Fibronectin.


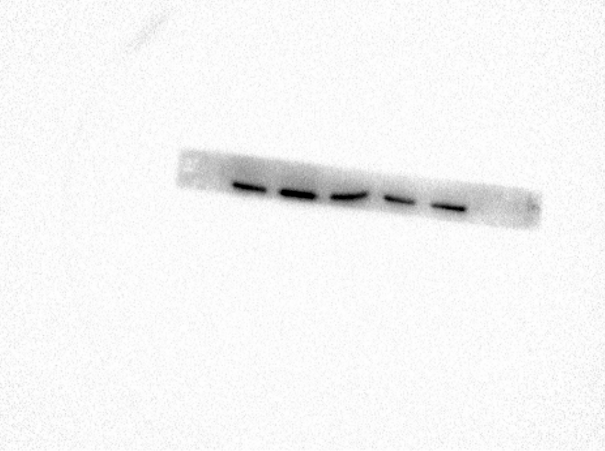


Figure 5: Vimentin.

3. Original western blot pictures of the third blots in figure3, the sequence of band from left to right is Normal group, Model group, Metformin group, DPDS-L group and DPDS-H group.


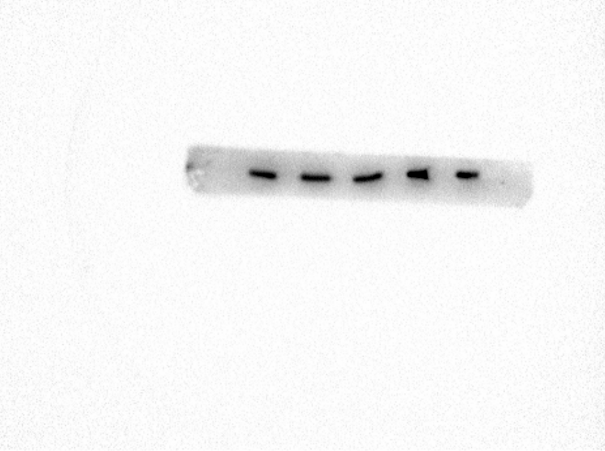

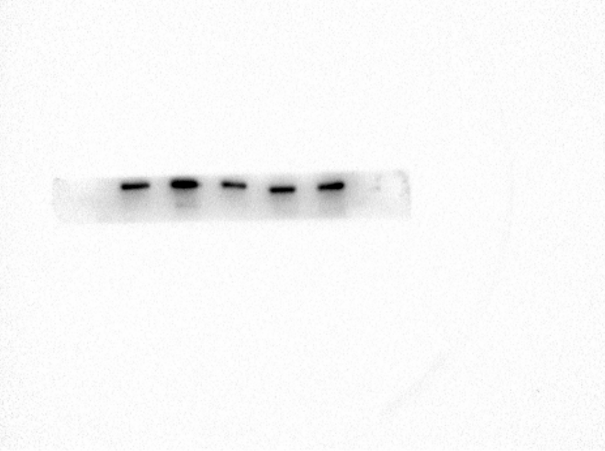


Figure 1: Actin. Figure 2: α-SMA.


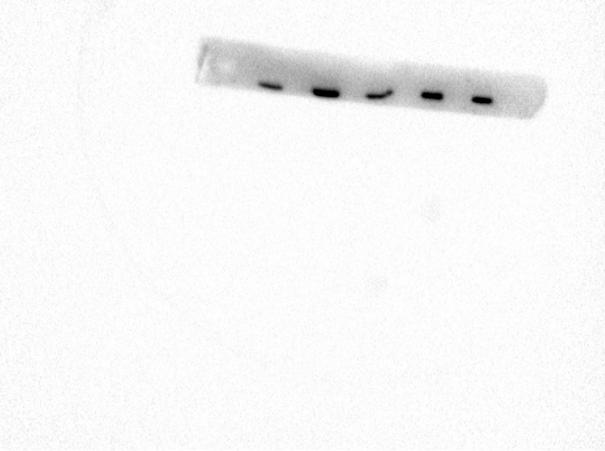

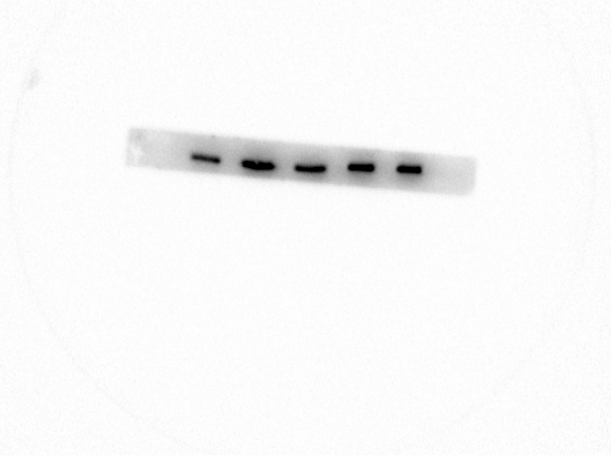


Figure 3: Collagen IV. Figure 4: Fibronectin.


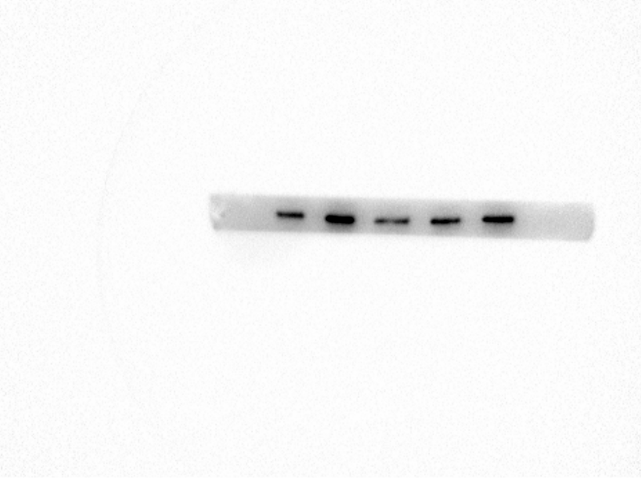


Figure 5: Vimentin.

4. Original western blot pictures of the fourth blots in figure3, the sequence of band from left to right is Normal group, Model group, Metformin group, DPDS-L group and DPDS-H group.


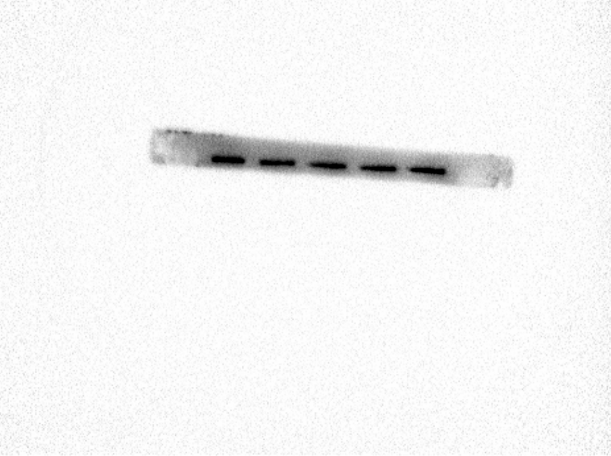

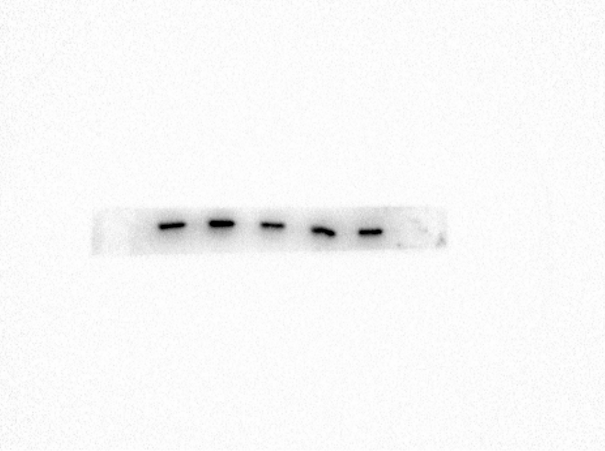


Figure 1: Actin. Figure 2: α-SMA.


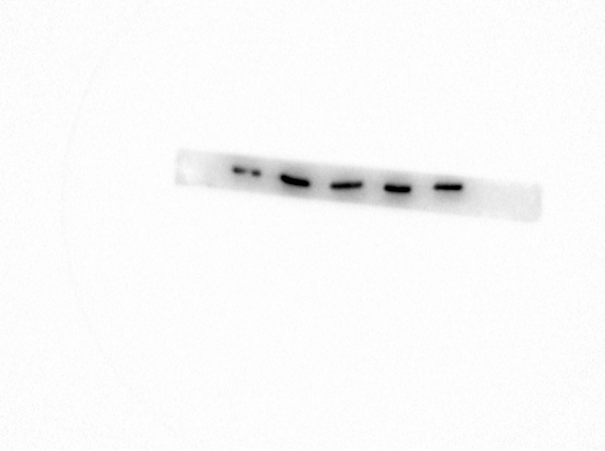

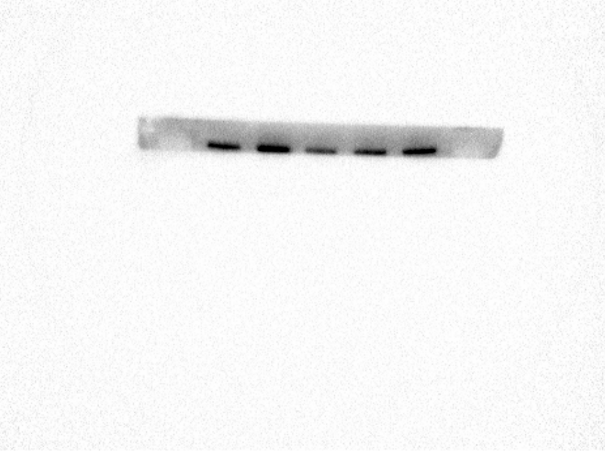


Figure 3: Collagen IV. Figure 4: Fibronectin.


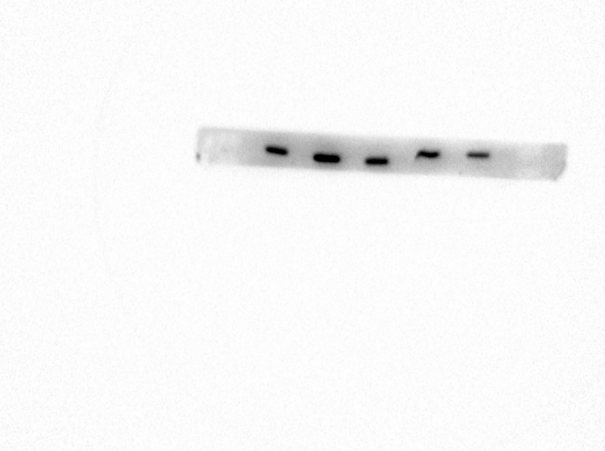


Figure 5: Vimentin.

1. Original western blot pictures of the first blots in figure4, the sequence of band from left to right is Normal group, Model group, Metformin group, DPDS-L group and DPDS-H group.


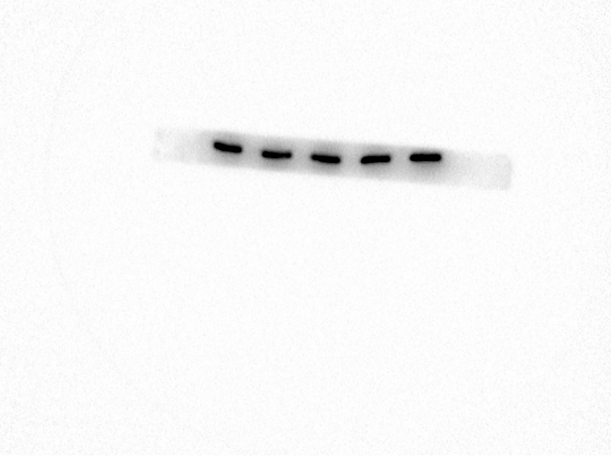

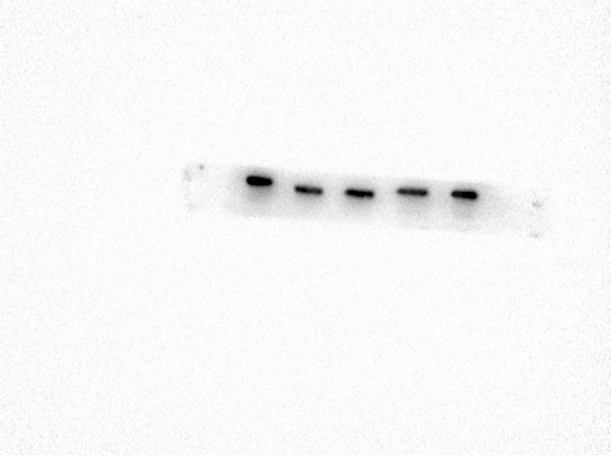


Figure 1: Actin. Figure 2: Nrf2.


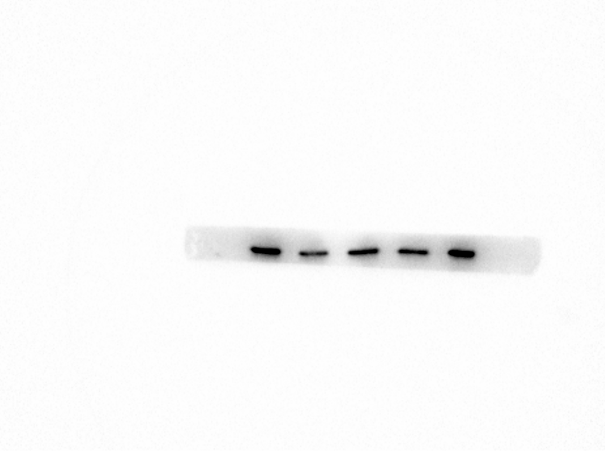

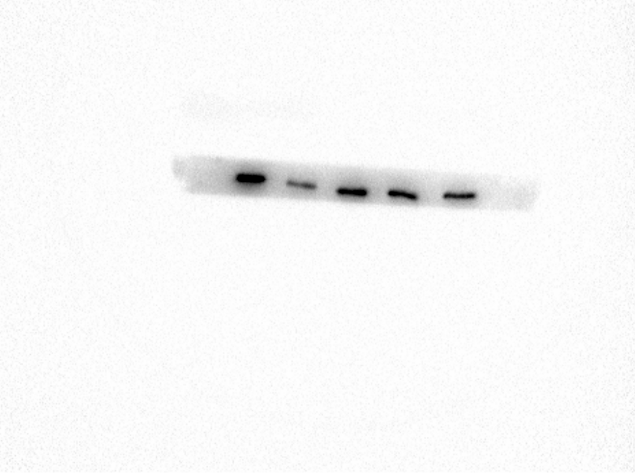


Figure 3: NQO1. Figure 4: HO-1.


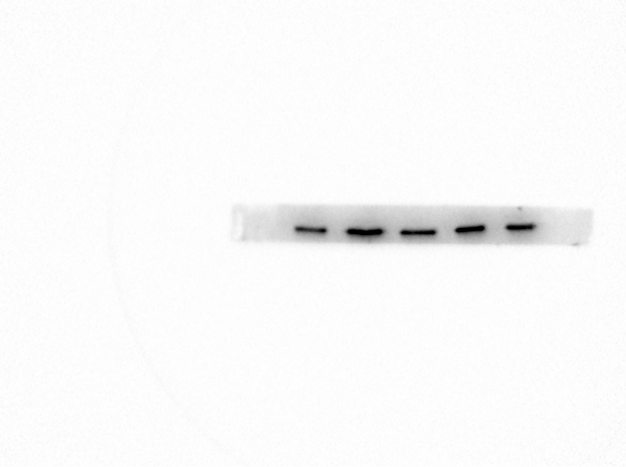


Figure 5: Keap1.

2. Original western blot pictures of the second blots in figure4, the sequence of band from left to right is Normal group, Model group, Metformin group, DPDS-L group and DPDS-H group.


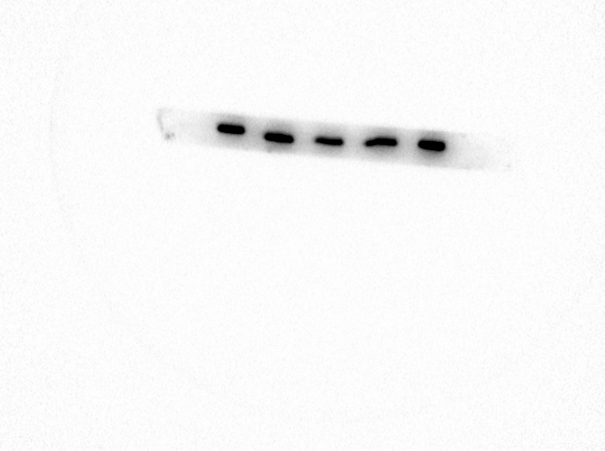

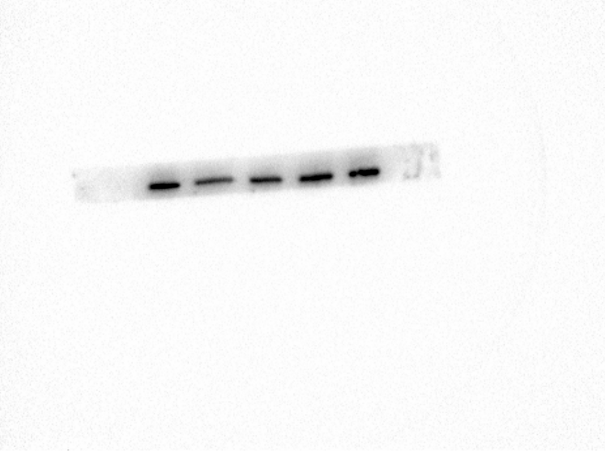


Figure 1: Actin. Figure 2: Nrf2.


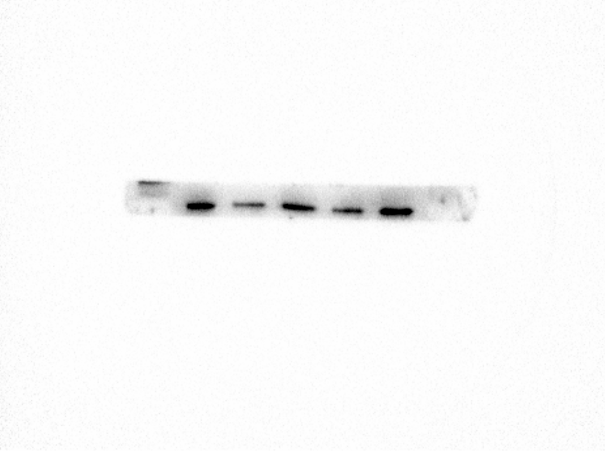

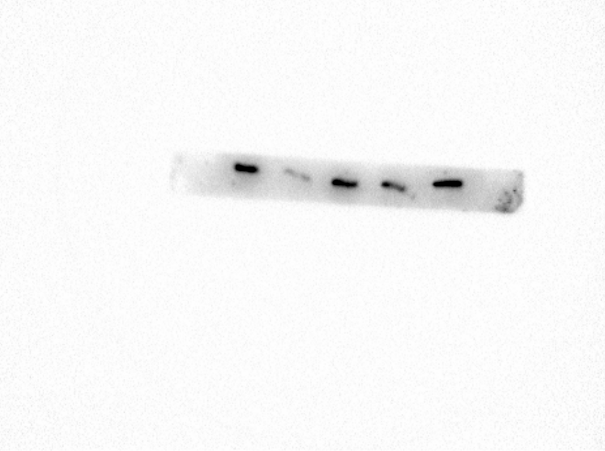


Figure 3: NQO1. Figure 4: HO-1.


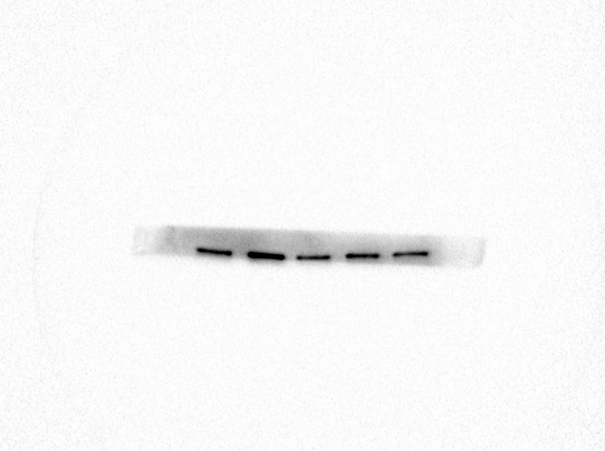


Figure 5: Keap1.

3. Original western blot pictures of the third blots in figure4, the sequence of band from left to right is Normal group, Model group, Metformin group, DPDS-L group and DPDS-H group.


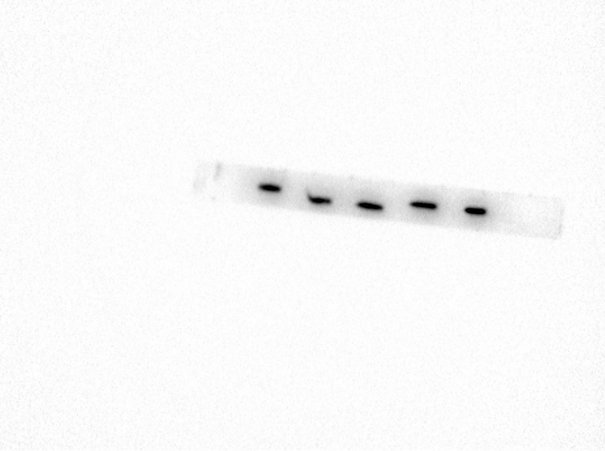

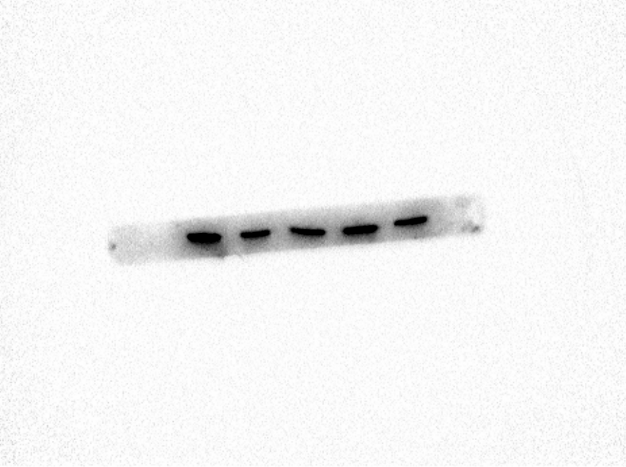


Figure 1: Actin. Figure 2: Nrf2.


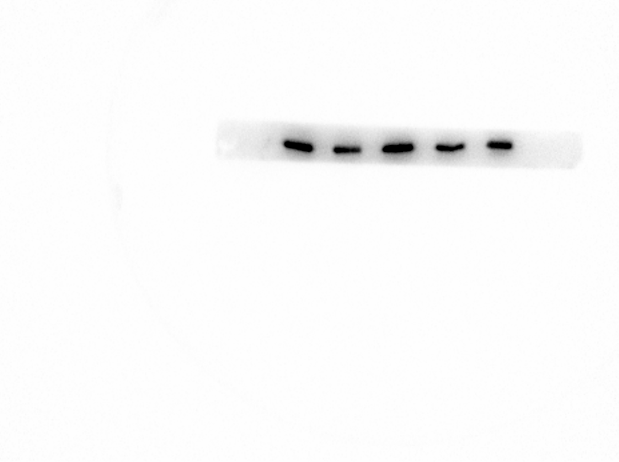

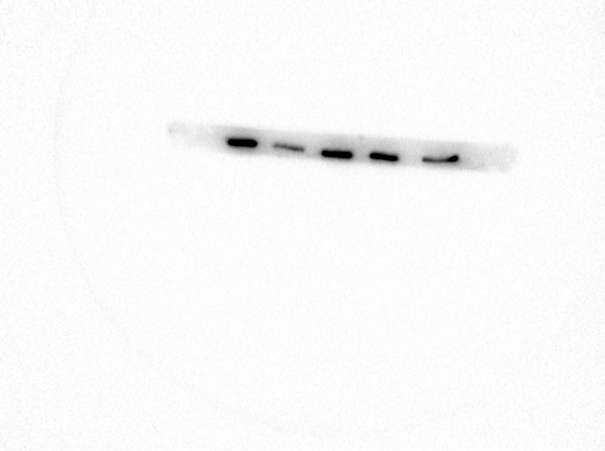


Figure 3: NQO1. Figure 4: HO-1.


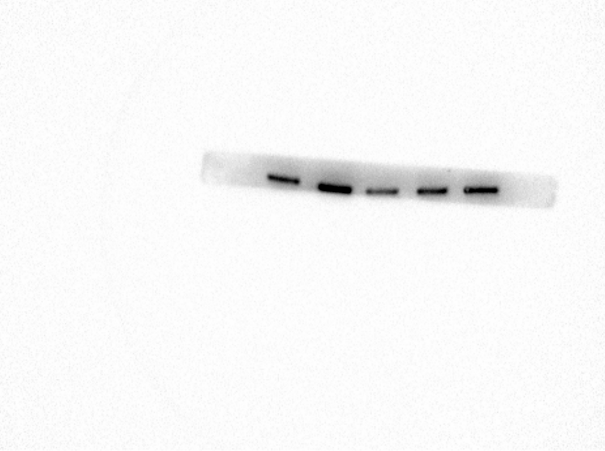


Figure 5: Keap1.

4. Original western blot pictures of the fourth blots in figure4, the sequence of band from left to right is Normal group, Model group, Metformin group, DPDS-L group and DPDS-H group.


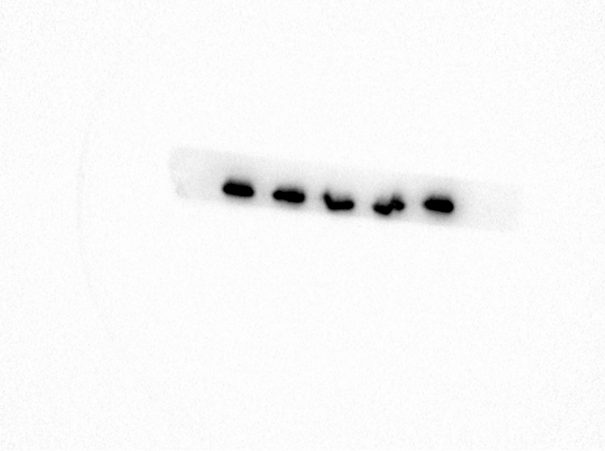

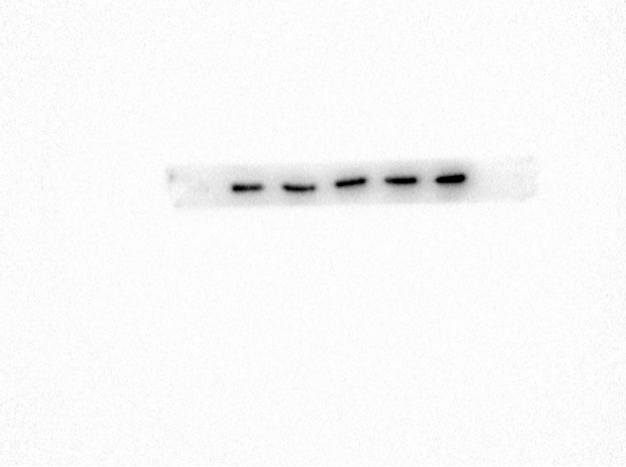


Figure 1: Actin. Figure 2: Nrf2.


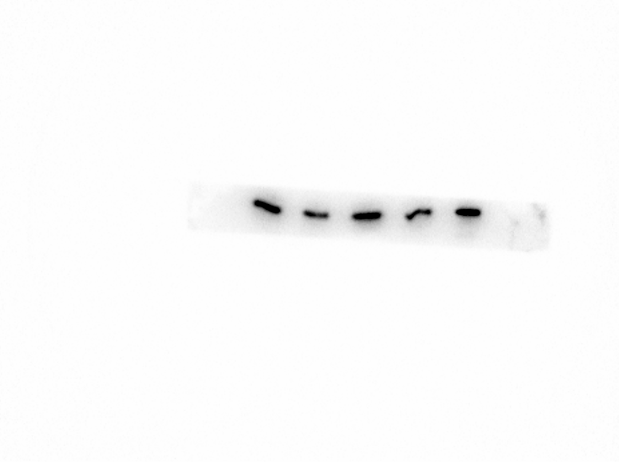

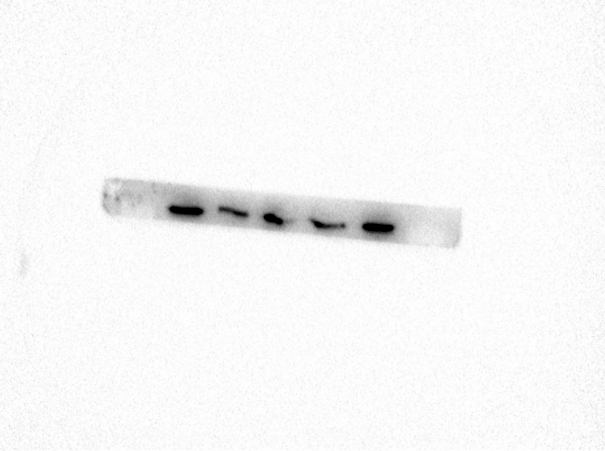


Figure 3: NQO1. Figure 4: HO-1.


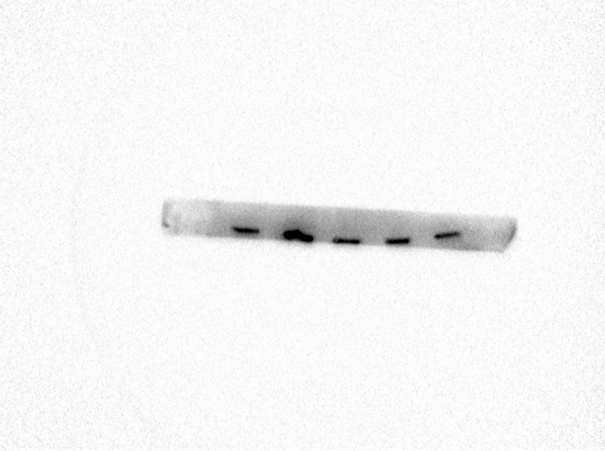


Figure 5: Keap1.
